# A chemoenzymatic synthesis of amide-containing quinazolin-4(*3H*)-one derivatives

**DOI:** 10.1039/d6ra02193j

**Published:** 2026-07-02

**Authors:** Mudzuli M. Maphupha, Marushka Soobben, Charles B. de Koning, Dean Brady

**Affiliations:** a Molecular Sciences Institute, School of Chemistry, University of the Witwatersrand PO Wits 2050 Johannesburg South Africa mudzuli.maphupha@wits.ac.za; b Wits Industrial Biotechnology Consortium, School of Molecular and Cell Biology, Faculty of Science, University of the Witwatersrand Johannesburg 2050 South Africa

## Abstract

The quinazolinone moieties are common in bioactive molecules, and novel variations of these compounds, preferably generated by sustainable methods, are of interest. Incorporating biocatalysis into the synthesis of active pharmaceutical ingredients offers significant advantages, mainly because enzymes have excellent specificity. In this work, we demonstrate the synthesis of flexible amide-containing quinazolin-4(*3H*)-ones from aminonitriles using a chemoenzymatic approach. We catalysed the hydration of aminonitriles to aminoamides using nitrile hydratase as a whole-cell biocatalyst. Subsequently, the aminoamides were coupled with ester-bearing benzaldehydes *via* microwave-assisted cyclisation, followed by laccase/DMSO-mediated oxidative aromatisation to afford the corresponding quinazolinone esters. The esters were then hydrolysed to their corresponding acids with immobilised lipase CALB (Novozym 435). Finally, lipase or 2-(1H-benzotriazol-1-yl)-1,1,3,3-tetramethyluronium hexafluorophosphate (HBTU) was used to carry out the amidation of the acid products, yielding the desired amide-containing quinazolin-4(*3H*)-one derivatives in excellent yields.

## Abstract 2

The quinazolinone moieties are common in bioactive molecules, and novel variations of these compounds, preferably generated by sustainable methods, are of interest.

## Graphical abstract

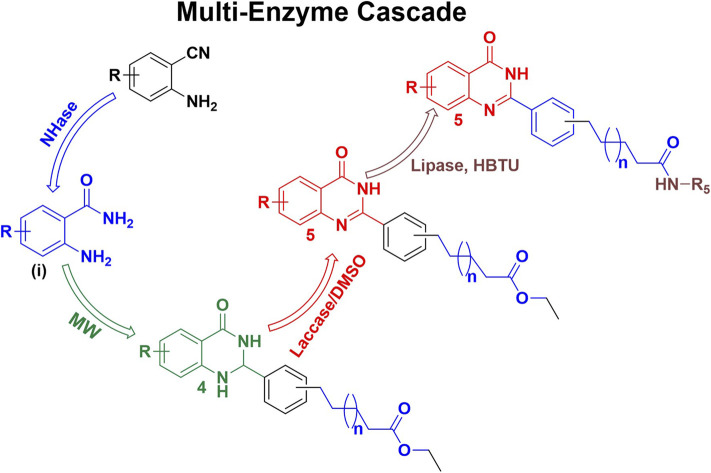

## Introduction

The development of greener chemical processes has become increasingly important in recent years, with growing emphasis on the use of safe catalysts, waste minimisation, replacement of toxic solvents, and application of environmentally benign oxidants.^1–3^ In this context, enzymatic heterocycle synthesis and multi-enzyme cascade strategies have advanced rapidly, supported by developments in protein engineering and chemoenzymatic integration.^2,4,5^ These approaches enable the stereoselective, modular, and scalable synthesis of complex N-, O-, and S-containing heterocyclic scaffolds, making them highly valuable for drug discovery and sustainable chemical manufacturing.^6–10^ Among the biocatalysts employed, laccases, nitrile hydratases, and lipases have emerged as powerful synthetic tools, offering high selectivity and catalytic efficiency while broadening the scope of green chemistry in heterocycle synthesis.^11–13^

**Fig. 1:**
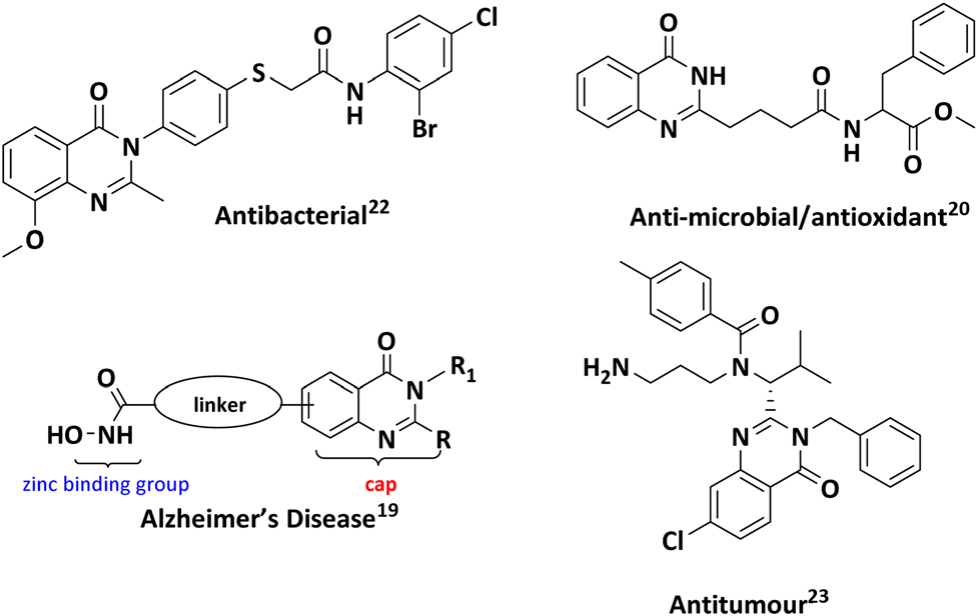
Bioactive quinazolinone compounds.

Quinazoline is a bicyclic aromatic heterocycle made up of a benzene ring fused to a pyrimidine.^14–16^ The quinazoline core exists in more than 100 alkaloids, generally present as quinazolin-4(*3H*)-one.^17,18^ It is an important pharmacophore, and its derivatives have been synthesised for a range of medicinal functions.^17^ Conjugating heterocycles such as quinazolinones to other bioactive moieties has been proven to be very effective.^19–21^ Notably, quinazolinones bearing amide functionalities have emerged as promising candidates, exhibiting a range of biological activities such as antibacterial,^22^ antitumour,^23^ antioxidant/antimicrobial,^20^ and anti-Alzheimer's disease^19^(Fig. 1). Other properties include anti-tumour,^24^ antituberculosis,^25^ analgesic,^26^ anti-inflammatory,^27^ antihypertensive,^28^ anticonvulsants,^29^ antimicrobial,^30^ antimalarial,^31^ antifungal,^31,32^ anti-viral,^33^ and antibiotic properties.^34^

Given the broad biological relevance of quinazolinones, there is sustained interest in developing efficient and sustainable methods for their synthesis and functionalization.^35,36^ However, conventional syntheses of quinazolinone derivatives often rely on multi-step protocols involving harsh conditions, prolonged reaction times, and toxic or non-renewable oxidants.^37,38^ A commonly used approach involves a three-component reaction between isatoic anhydrides, 2-aminobenzamides, and aldehydes, followed by oxidation of the resulting intermediate.^3^ However, the oxidants typically used in these transformations—such as MnO_2_,^39^ TBHP,^40^ FeCl_3_,^41^ Cu(OAc)_2_,^42^ I_2_,^43^ NaHSO_3_,^44^ and KMnO_4_ ^45^—are associated with poor atom economy, environmental concerns, and hazardous waste generation.

Despite advances in synthetic methodologies, achieving high chemo-/regioselectivity and functional group diversity through green practices remains a key challenge.^46^ In particular, selective quinazolinone formation using sustainable oxidants and mild conditions is still limited.^47,48^ To address these limitations, cascade and tandem strategies have emerged as economical routes to quinazolinones from alcohols, aldehydes, *o*-aminobenzamides, and aminobenzylamines.^49–52^ Recent efforts increasingly favour biocatalytic systems, such as lipases and laccases, to replace harsh chemical steps.^38,53^ However, existing enzymatic approaches often suffer from narrow substrate scope, poor compatibility between reaction steps, and limited scalability.^54,55^

Herein, we directly address these gaps by introducing a tandem chemoenzymatic protocol for the synthesis of quinazolin-4(3*H*)-one derivatives bearing amide functionalities. This work focuses on synthesising the quinazolinone core and incorporating a flexible amide functionality to generate hybrid compounds with potentially improved pharmacokinetic properties and bioavailability. This will be achieved through a chemoenzymatic strategy involving three complementary biocatalysts and selected chemical transformations. First, nitrile hydratases will be employed to replace conventional nitrile hydration, avoiding strong acids/bases, high temperatures, and metal catalysts while enabling direct access to 2-aminobenzamides under mild aqueous conditions.^56,57^ The resulting intermediates will then undergo microwave-assisted condensation/cyclisation to generate 2,3-dihydroquinazolin-4(1*H*)-one derivatives within short reaction times, reducing the energy demand typically associated with conventional heating. Subsequent laccase/DMSO-mediated oxidation, coupled with molecular oxygen as a green oxidant, facilitates selective oxidative cyclisation without the use of stoichiometric oxidants or toxic mediators.^52,58,59^ Immobilised *Candida antarctica* lipase B (CALB, Novozym 435) enables efficient late-stage functionalisation in organic media, overcoming the limitations of free enzymes and enhancing catalyst stability.^13,53^ Finally, HBTU-mediated amidation will be used to afford the target quinazolinone amides in excellent yields. To the best of our knowledge, no biocatalytic protocol has yet been reported that enables the synthesis of quinazolinone scaffolds with the incorporation of amide functionalities.

## Results and discussion

**Scheme 1:**
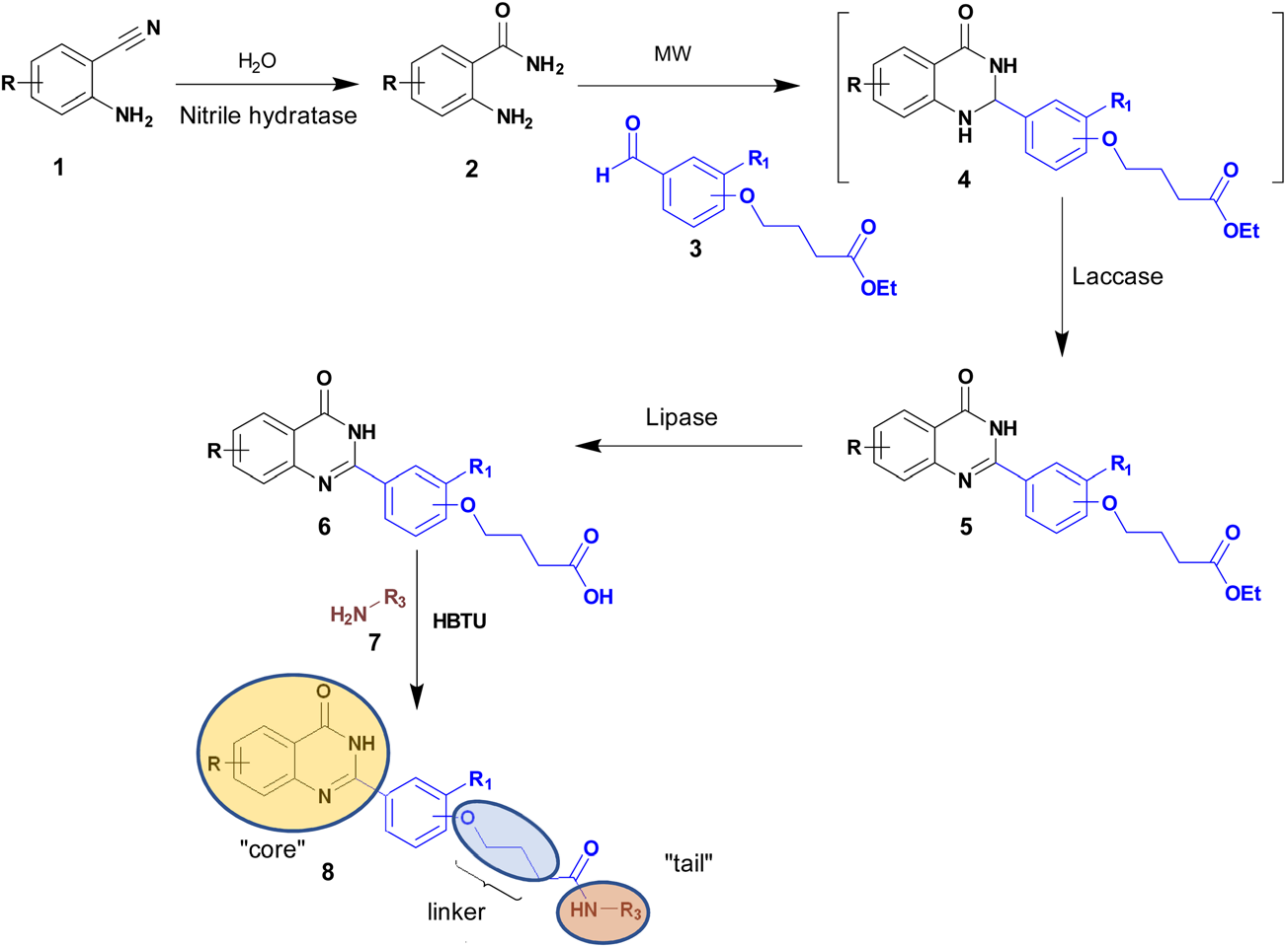
Chemoenzymatic route for the synthesis of quinazolin-4(*3H*)-one derivative containing an amide moiety.

As shown in Scheme 1, the quinazolinone series is achieved through a five-step chemoenzymatic sequence that combines two chemical transformations with three biocatalytic steps: (i) nitrile hydration of amino nitrile 1 to the corresponding amide 2, catalysed by nitrile hydratase. (ii) A microwave-assisted condensation/cyclisation generates 2,3-dihydroquinazolin-4(*1H*)-one 4. (iii) A laccase-catalysed oxidative dehydrogenation aromatises 4 to the corresponding quinazolinone ester 5. (iv) Hydrolysis of the subsequent quinazolinone ester 5 mediated by lipases. (v) HBTU-mediated amidation converts 5 into the final quinazolinone amide product 8.

### Synthesis of 2-aminobenzamides

**Scheme 2:**
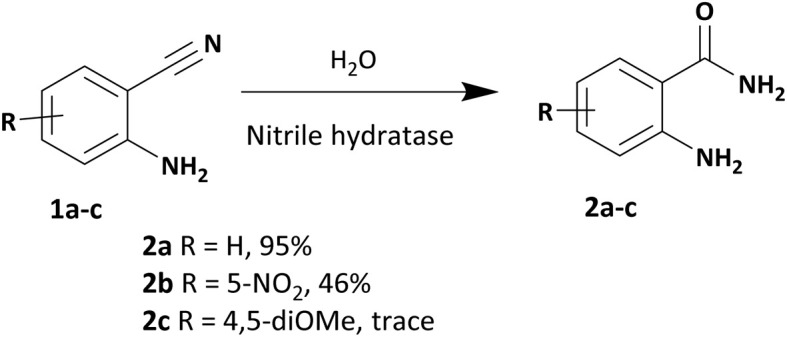
Nitrile hydratase-catalysed synthesis of 2-aminobenzamide derivatives.

Quinazolin-4(*3H*)-one derivatives were synthesised from the reaction of 2-aminobenzamides and aldehydes under aerobic conditions. Initially, 2-aminobenzamides were prepared by hydrating nitriles 1a–c using nitrile hydratases according to Brady *et al.* (2004)^60^ (Scheme 2).

A series of benzonitrile derivatives was reacted with 50.0 mL cell-free extract (*R. rhodochrous* ATCC BAA-870) with a measured nitrilase activity of 32 U (determined by the conversion of 960 µmol of benzonitrile to benzoic acid in 30 min).^61^ In a round-bottom flask, 50 mL of NHase-containing cell lysate was added to a solution of 2-aminobenzonitrile derivatives (10.0 mmol) in 18 mL of Tris buffer (pH 7.6) and 10% acetone (v/v). The reaction was maintained at 25 °C under constant stirring for 24 h.

The nitrile hydratase preparation showed low affinity for 2-amino-4,5-dimethoxybenzonitrile 1c. Monitoring by TLC showed low conversions (<5%), with the reaction mixture primarily consisting of unreacted substrate. In contrast, overall activity toward 2-aminobenzonitrile and its 5-nitro derivative was satisfactory under the conditions tested. Based on these outcomes, no further optimisation of the reaction parameters was undertaken.

### Synthesis of 2,3-dihydroquinazolin-4(*3H*)-one derivatives

**Scheme 3:**
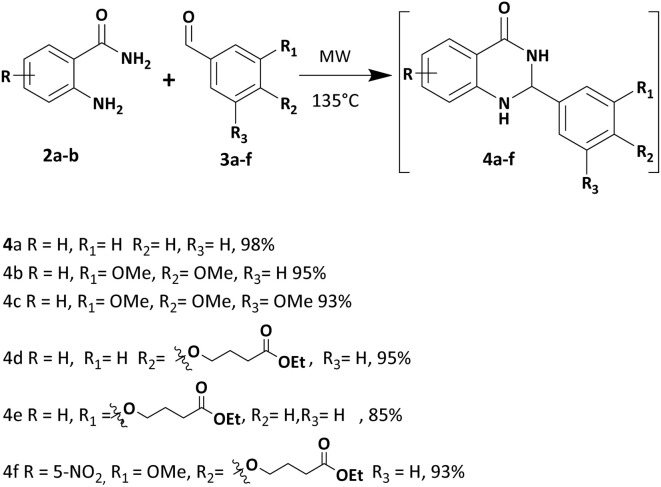
Microwave-assisted laccase cyclisation synthesis of quinazolin-4(1*H*)-ones.

**Scheme 4:**
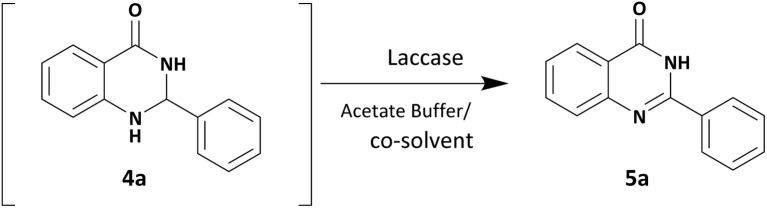
Oxidation of 2-phenylquinazolin-4(1*H*)-one.

The oxidative cyclisation to form quinazolin-4(3*H*)-one was carried out in two steps. Firstly, 2-aminobenzamides 2a–b were condensed with benzaldehyde derivatives 3a–f under microwave irradiation to yield 2,3-dihydroquinazolin-4(1*H*)-one **4a–f** (Scheme 3). Next, the 2,3-dihydroquinazolin-4(1*H*)-one derivatives were oxidised using a laccase enzyme under aerobic conditions, thereby introducing a double bond to afford the target quinazolinone derivatives (Scheme 4).

### Microwave-assisted cyclocondensation

The cyclocondensation reaction of benzamides 2a–b with aldehydes 3a–f to form 2,3-dihydroquinazolin-4(1*H*)-one 4 was carried out under microwave-assisted, solvent-free conditions. Microwave-assisted cyclisation of anthranilamide and aldehydes has been reported to be highly efficient in the literature.^62^ Bakavoli *et al.* (2018) reported a microwave-assisted synthesis of quinazolin-4(3*H*)-ones using potassium permanganate in dimethylacetamide as an oxidant, producing high yields of products.^63^ Kang *et al.* (2018) reported another efficient microwave-assisted synthesis of quinazolin-4(3*H*)-ones using antimony(iii) trichloride (SbCl_3_) as a Lewis acid catalyst.^64^

Benzaldehydes 3d–f were prepared from hydroxy-benzaldehydes *via* the Williamson ether method in acetone.^65^ We found that, under conventional heating conditions, benzaldehydes 3d–f tend to decompose during condensation with aminoamides 2. However, this was not observed using microwave irradiation. The cyclocondensation of 2 and 3 afforded the desired 2,3-dihydroquinazolin-4(1*H*)-ones 4 with quantitative conversions within 5 minutes (Scheme 3).

### Laccase-catalysed aerobic oxidative dehydrogenation

After establishing an efficient microwave-assisted method for the cyclocondensation of benzamide 2 and aldehyde 3, we investigated the aerobic oxidation of 2,3-dihydroquinazolin-4(1*H*)-ones 4 using laccase. The oxidation of 2-phenylquinazolin-4(1*H*)-one 4a to 2-phenylquinazolin-4(3*H*)-one 5a was chosen as the model reaction. Reaction parameters, including solvents/co-solvents, temperature, and mediators, were investigated. The oxidations were performed using laccase in an acetate buffer (0.1 M, pH 4.0) and various co-solvents (75% v/v) (Scheme 4 and Table 1). A commercially available immobilised laccase (Novoprime Base 268) was selected due to its high thermal stability.^66^

**Table 1:** Novoprime Base 268-catalysed oxidation of 2-phenylquinazolin-4(1*H*)-one 4a in the presence of different mediators and co-solvents

Entry	Laccase	Mediator	Co-solvent (75% v/v)	Temperature (°C)	Time (h)	Yield (%)
1	Control reaction	—	—	100	24	0
2	Control reaction	—	DMSO^a^	70	36	30
3	Control reaction	—	DMSO	120	48	96
4	Novoprime base 268	—	—	70	24	35
5	Novoprime base 268	—	DMSO	70	18	95
6	Novoprime base 268	—	Acetonitrile	70	24	30
7	Novoprime base 268	—	DMF	70	18	31
8	Novoprime base 268	ABTS	Methanol	70	24	0
9	Novoprime base 268	TEMPO	Methanol	65	24	0
10	Novoprime base 268	TEMPO	Ethanol	65	18	0
11	Novoprime base 268	ABTS	Acetonitrile	70	24	55
12	Novoprime base 268	ABTS	DMF	70	24	57
13	Novoprime base 268	ABTS	DMSO	70	24	95
14	Novoprime base 268	TEMPO	DMSO	70	16	97

a The reaction was carried out with pure DMSO, without a buffer.

Initially, the reaction was carried out in the absence of enzyme or mediator, and no conversion of the substrate was observed (Table 1, entry 1). When laccase was introduced into the reaction medium, a low yield was observed in an acetate buffer (Table 1, entry 4). Co-solvents and mediators were included to improve conversion and substrate solubility. However, methanol and ethanol appear to have deactivated the enzyme (Table 1, entries 8, 9, 10), and neither acetonitrile nor DMF improved yields (Table 1, entries 6, 7), although the presence of the mediator ABTS (Table 1, entries 11, 12) did have a positive effect.

However, DMSO in buffer afforded excellent yields (Table 1, entries 3, 5, 13, 14), even in the absence of the catalyst. DMSO is known to oxidise quinazolinones. We compared our laccase/DMSO reaction with the reported DMSO method (DMSO/100°C/36h/98% yield)^67^ for the oxidation of 2-phenyl-2,3-dihydroquinazolin-4(1*H*)-one 4. Incorporating laccase significantly sped up the reaction (from 36 h to 18 h) and simultaneously reduced the required reaction temperature from 100 °C to 70 °C (Table 1, entry 5), and the omission of the mediator did not have an observable impact.

Interestingly, only limited conversions were observed in undiluted DMSO at 70 °C (Table 1, entry 2). Both Shariati *et al.* (2019)^68^ and Heidary *et al.* (2014)^69^ reported loss of activity for the oxidation of quinazolines in pure acetonitrile, THF, DMF, and MeCN, probably due to enzyme denaturation, while using soluble enzyme. Shariati *et al.* also observed the same loss of activity in DMSO; however, in contrast, immobilised Novoprime Base 268 maintained activity in high DMSO concentrations. Laccase solvent tolerance is highly dependent on enzyme origin, and substantial activity has been reported at elevated solvent fractions.^70,71^*Poliporus pinsitus* laccase retained ≥70% activity in mixtures containing up to 60% (w/w) dioxane, isopropanol, ethylene glycol, or acetonitrile, although activity decreased within 24 h in most cases.^70^ Similarly, the directed-evolved fungal variant MtTL2 R2 maintained >80% activity after 24 h in 50% (v/v) acetonitrile, dimethylacetamide, dimethylformamide, DMSO, acetone, methanol, or ethanol.^72^

**Scheme 5:**
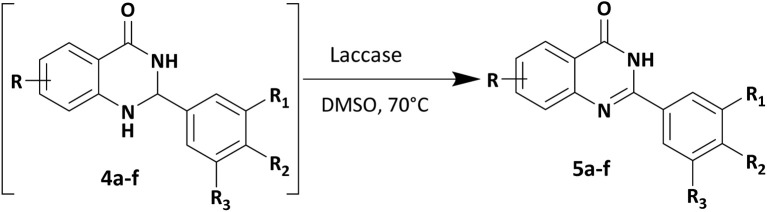
Oxidation of 2,3-dihydroquinazolin-4(1*H*)-ones.

After establishing an effective DMSO/laccase-catalysed aerobic synthesis of 2,3-dihydroquinazolin-4(3*H*)-one, we tested its applicability across various benzaldehyde substrates (Table 2, 4a–f). Specifically, we wanted to determine whether this procedure could be effective with substrates bearing ester chains (Table 2, entries 4-6), as steric hindrance from longer chains could potentially limit substrate access to the enzyme's active site (Scheme 5).

**Table 2:** A laccase/DMSO catalysed aerobic oxidative synthesis of 2-substituted quinazolin-4(3*H*)-one derivatives

Entry	Substrate	Product 5a–f	Time (h)	Yield (%)
1	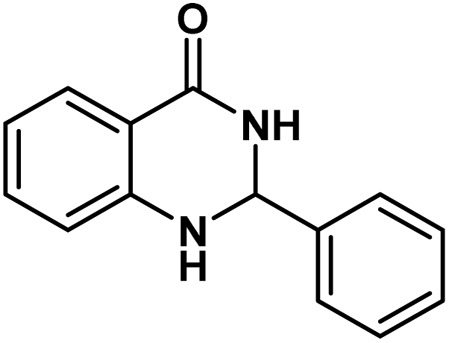	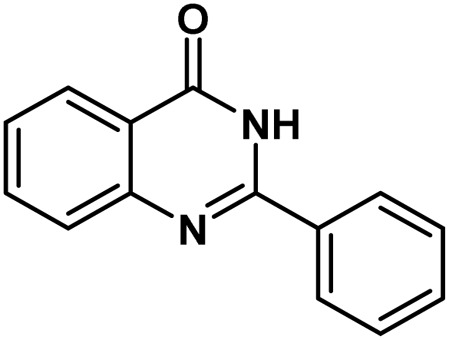	18	95
2	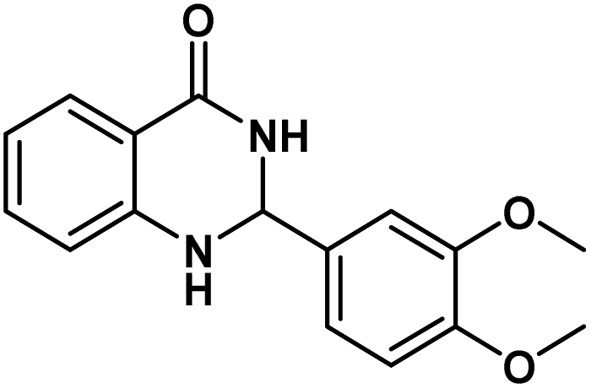	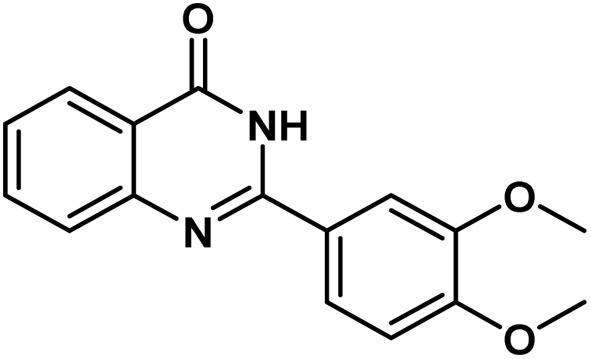	16	99
3	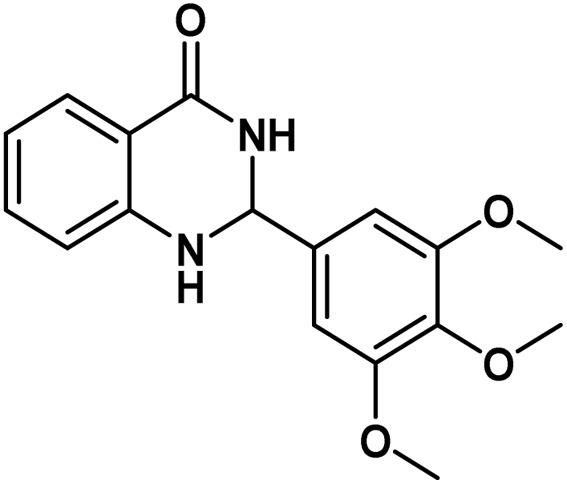	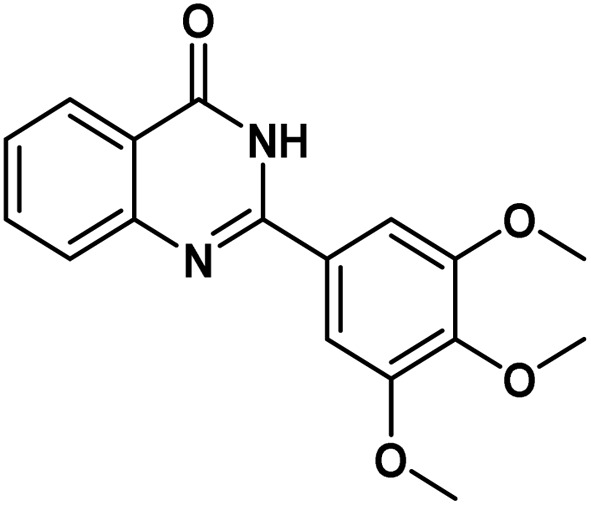	16	98
4	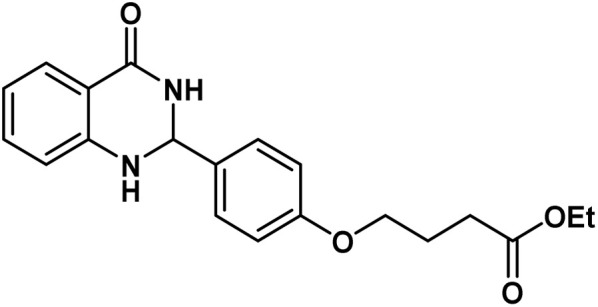	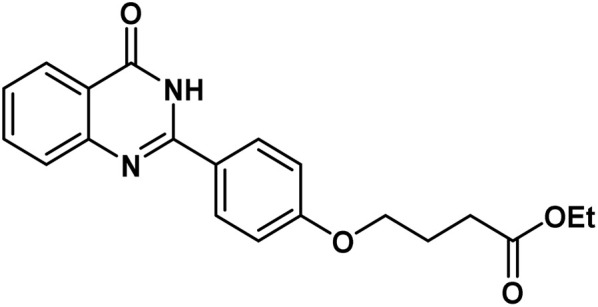	18	96
5	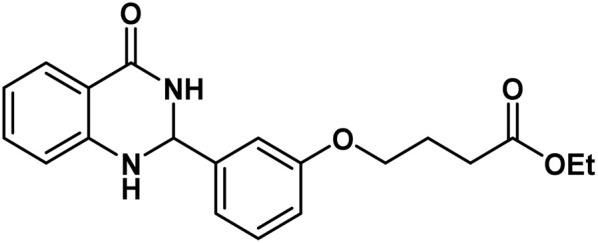	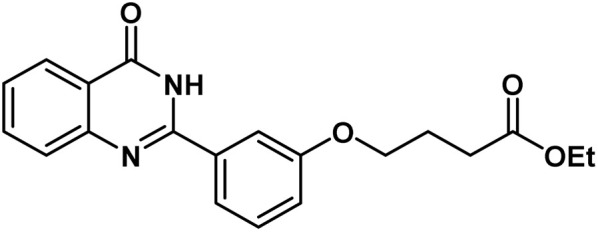	18	90
6	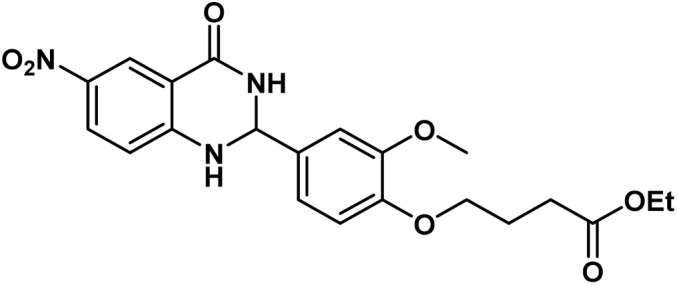	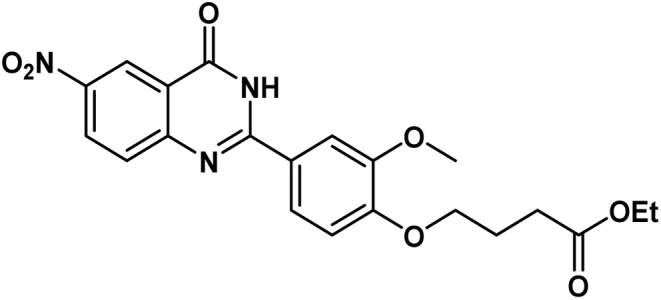	18	99

The expected reaction proceeded efficiently to afford 2,3-disubstituted quinazolinone esters in excellent yields (Table 2, entries 4-6). The DMSO-laccase (Novoprime Base 268) proved to be effective for the oxidative dehydrogenation of 2,3-dihydroquinazolin-4(*1H*)-ones across a broad substrate range.

**Scheme 6:**
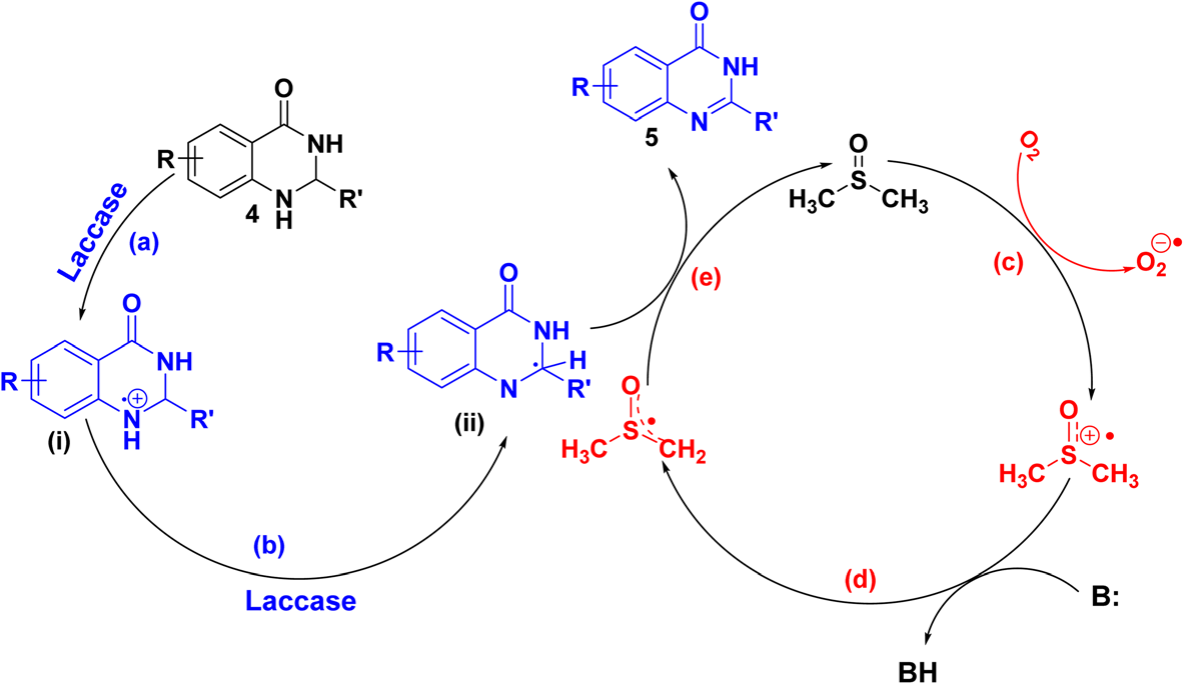
Proposed mechanism for the synthesis of quinazolinones using laccase/DMSO system.

Currently, the exact reaction mechanism of the laccase/DMSO oxidation of 2,3-dihydroquinazolin-4(1*H*)-one has not been established and requires further study. However, based on literature reports, we propose that the laccase/DMSO-mediated oxidation proceeds through an electron-transfer, radical-chain pathway, in which laccase and DMSO act cooperatively to achieve the oxidised product 5 (Scheme 6). In this scenario, two interconnected radical-generating systems occur concurrently: (1) laccase-mediated two-electron oxidation of the substrate to achieve key substrate radicals, and (2) aerobic oxidation of DMSO to produce reactive radical species (aerobic oxidation of DMSO mechanism was adopted from Lin *et al.* (2023)).^73^ Subsequent radical–radical coupling/termination between these species is then proposed to achieve the target product.

Computational studies suggest that for the oxidation of 4 to 5, intermediates (i) and (i) are expected along the radical-chain sequence (Scheme 6).^74,75^ First, laccase promotes initial single-electron transfer (SET) from the heterocycle, plausibly through electron abstraction at N1, to give the radical intermediate (i) (step a). The azabenzyl radical cation is stabilised through extensive delocalisation of the radical centre across the adjacent aromatic system.^75^ A second laccase-mediated oxidation coupled with proton loss is then proposed to generate a more stabilised benzylic (donor-substituted) radical species (ii) (step b). The radical centre is stabilised through delocalisation across the C2-positioned aromatic ring, complemented by electronic interactions with the lone pairs of the N1 nitrogen atom.^75^ In parallel, DMSO reacts with O_2_ to form a DMSO radical cation (DMSO˙^+^) together with the highly reactive superoxide radical anion (O_2_˙^−^) (step c).^73^ Deprotonation of the newly formed DMSO˙^+^ by a Lewis base present in the reaction mixture (anthranilamide in this system) is then proposed to yield a neutral DMSO-derived radical (step d). Finally, coupling (or hydrogen transfer followed by oxidation) between the substrate-centred radical (ii) and the DMSO-derived radical is proposed to achieve the final oxidised product 5. Overall, DMSO therefore plays multiple roles in the transformation, serving not only as a solvent, radical mediator and redox participant throughout the reaction.

### Lipase-catalysed direct amidation of esters 5d–f

**Scheme 7:**
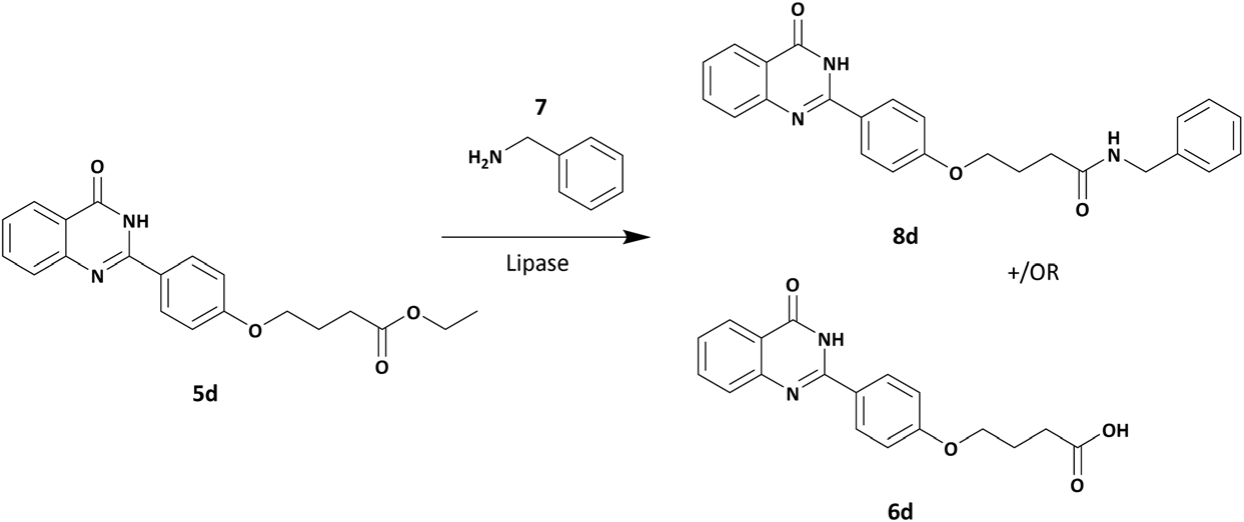
The amidation of quinazolinone esters with amines under lipase conditions.

Subsequently, we investigated a lipase-catalysed direct amidation of the obtained quinazolinone esters 5 with amines under mild conditions. Immobilised CALB was selected for its broad substrate range and its compatibility with organic solvents.^76^ As a model reaction, ethyl 4-(4-(4-oxo-3,4-dihydroquinazolin-2-yl)phenoxy)butanoate (5d) was coupled with benzyl amine using Novozymes immobilised *Candida antarctica* Lipase B (CALB) in various solvents (Table 3 and Scheme 7).

**Table 3:** CALB catalysed amidation of ethyl 4-(4-(4-oxo-3,4-dihydroquinazolin-2-yl)phenoxy)butanoate 5d with benzylamine

Entry	Ester	Solvent	Reaction time (days)	Product	
				8d	6d^a^
1	5d	Toluene	3	−	−
2	5d	DMF	3	−	40%
3	5d	DMSO	3	−	30%
4	5d	Acetonitrile	5	−	−
5	5d	2-MeTHF	5	−	−
6	5d	1,4-Dioxane	5	−	Trace amounts
7	5d	Acetone	5	−	Trace amounts
8	5d	Diethyl ether	5	−	−

a -Estimated conversion from crude ^1^H NMR.

Substrate solubility was a challenge in many of the solvents screened, particularly for ethyl 4-(4-(4-oxo-3,4-dihydroquinazolin-2-yl)phenoxy)butanoate 5d. However, improved solubility was observed in DMF and DMSO. To maintain enzyme stability, reactions were conducted in the presence of a small amount of water (10% v/v); however, this condition promoted hydrolysis rather than amidation (Table 3, Entries 2–3). None of the tested solvents supported the desired amidation reaction.

### Solvent screening for the lipase hydrolysis of ester 5

**Scheme 8:**
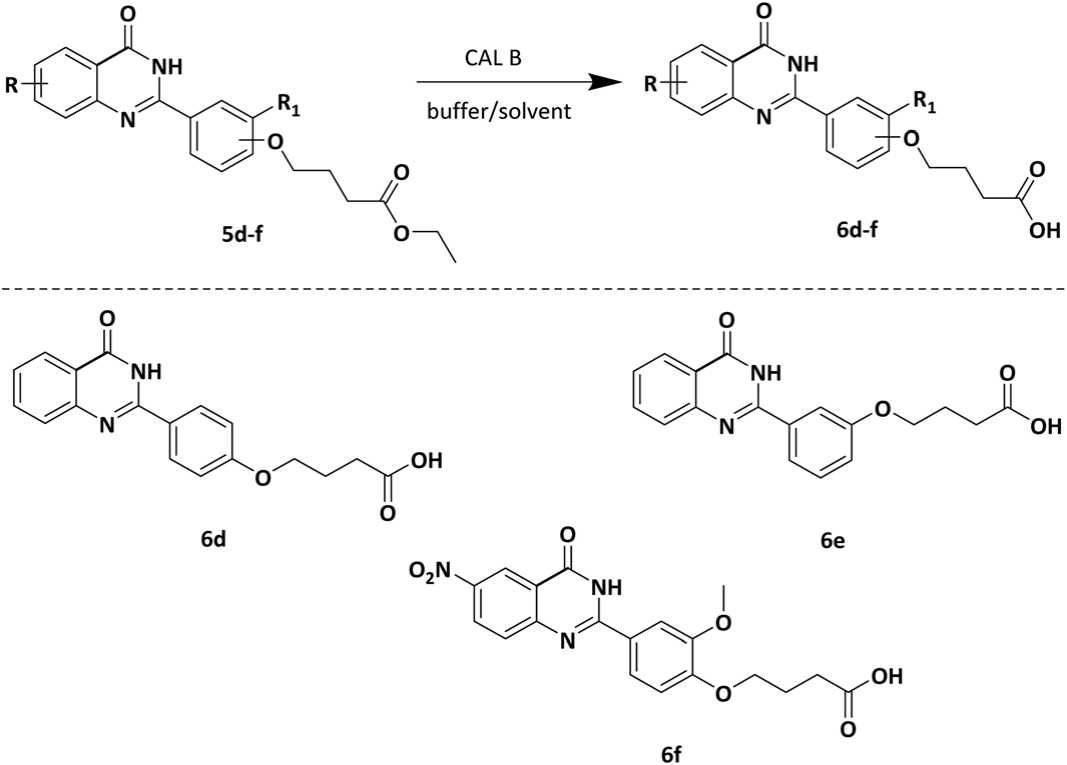
Novozym 435-catalysed hydrolysis of quinazolinone ester 5.

Because lipase-catalysed amidation of quinazolinone ester 5d resulted in the hydrolysis of the ester, we investigated the lipase-mediated hydrolysis of the three quinazolinone esters 5d–f to obtain the corresponding carboxylic acids 6d–f (Scheme 8).

The lipase reactions were carried out in a phosphate buffer (0.2 M, pH 7.4) with 70% DMSO (v/v) at 30 °C. Novozym 435 effectively hydrolysed quinazolinone esters 5d–f to their corresponding carboxylic acids, with DMSO chosen as a co-solvent to promote substrate solubility. We also screened greener solvents, including acetone and 2-MeTHF; however, this resulted in lower conversions due to limited substrate solubility. The overall trend was that yields over 80% were achieved with the dipolar aprotic solvents DMSO and DMF, reasonable yields (40–60%) were obtained with acetone, and poor to negligible conversions (<10%) occurred in 2-MeTHF, 1,4-dioxane, and acetonitrile (Table 4).

**Table 4:** Solvent screening for the hydrolysis of the three quinazolinone ester derivatives 5d–f

Entry	Ester	Solvent	Reaction time (days)	Yield 6 (%)
1	5d	DMF	1	82
2	5e	DMF	1	66
3	5f	DMF	1	52
4	5d	DMSO	1	80
5	5e	DMSO	1	65
6	5f	DMSO	1	68
7	5d	Acetone	5	56
8	5e	Acetone	5	48
9	5f	Acetone	5	51
10	5d	2-MeTHF	5	0
11	5e	2-MeTHF	5	0
12	5f	2-MeTHF	5	0
13	5d	1,4-Dioxane	5	0
14	5e	1,4-Dioxane	5	0
15	5f	1,4-Dioxane	5	0
16	5d	DMSO	3	86
17	5e	DMF	3	76
18	5f	DMSO	3	70

Substrate solubility plays a significant role in the outcome of the reaction. To optimise this step, we evaluated the effects of temperature, buffer pH, and reaction time. The best conditions were determined as 30 °C, pH 7.5, and reaction time of 72 h (3 days) (Table 4, entry 16).

### Lipase screening for amidation reactions

**Scheme 9:**
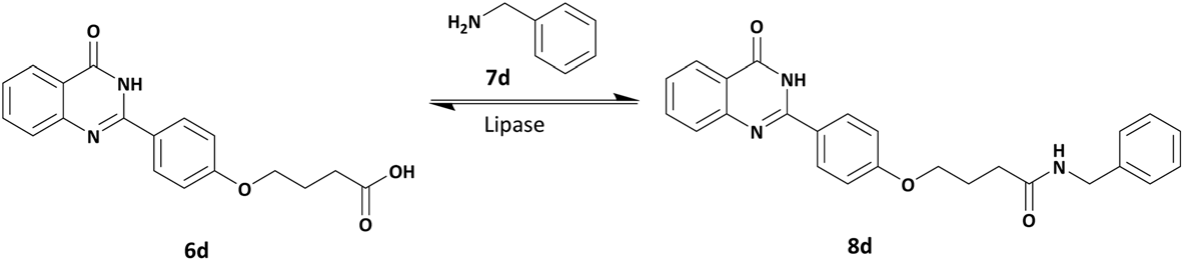
Lipase-catalysed amidation of quinazolinone acid 6.

Lipases are well-known for exhibiting a wide range of substrate specificities. We therefore investigated the effects of lipase origin and preparation on the amidation reaction of quinazolinone acids 6. The reaction between butanoic acid 6d and benzylamine 7a was chosen as a model reaction, and it was performed under anhydrous conditions in dry DMSO, DMF, and acetone with 3 Å molecular sieve (50 mg) to minimise moisture (Table 5 and Scheme 9).

**Table 5:** Lipase screening

Entry	Catalyst	Solvent	Temperature (°C)	Time (h)	Yield 8 (%)
1	—	DMF	100	72	—
2	Lipozyme CALB L (Novozymes)	Acetone	30	72	Trace
3	*P. fluorescens* lipase (Amano)	Acetone	30	72	—
4	Novozym 435	Acetone	50	72	65
5	Lipozyme CALB L (Novozymes)	DMSO	35	72	—
6	*P. fluorescens* lipase (Amano)	DMSO	30	72	—
7	Novozym 435	DMSO	50	72	Trace
8	Novozym 435	DMF	30	72	—
9	*P. fluorescens* lipase (Amano)	DMF	35	72	—
10	Lipozyme CALB L (Novozymes)	DMF	50	72	Trace
11	HBTU	DMF	25	2	95

As a positive control, the reaction was conducted without a catalyst in DMF, and no amide formation was observed. Lipases *P. fluorescens* (Amano; Merck Cat. No. 534730) and Lipozyme CALB L (Novozymes) were almost entirely inactive (<5% conversion) (Table 5, entries 2, 3, 5, 6, 9). In contrast, immobilised CALB (Novozym 435) in acetone afforded 65% yield of carboxylic amide 8d (Table 5, entry 4). Unfortunately, DMSO (and the other dipolar aprotic solvent DMF) was ineffective for amidation, promoting hydrolysis instead, possibly due to their hygroscopic nature and the requirement for low-water conditions in lipase amidation reactions (Table 5, entries 7, 8).

Immobilised CALB lipases, such as Novozym 435, have previously been shown to catalyse amidation reactions effectively.^77^ We screened a series of amines (Table 6) using Novozym 435 in acetone, as it demonstrated activity in initial screenings. Unfortunately, this method was ineffective for most substrates, with only one example achieving moderate conversion (Table 6, entry 1, 60% yield). The yields exhibited significant variability, frequently falling below the initial 65% observed during the preliminary enzymatic screening, likely due to inefficiencies in substrate–enzyme interaction. We suspect several factors may be limiting the reaction, including steric hindrance, reduced enzyme activity in the absence of water, and poor substrate solubility, the latter of which appears to be the major limitation. However, it is plausible that other lipases or other classes of enzymes may be able to extend the substrate range.

**Table 6:** HBTU-catalysed direct amidation of quinazolinone carboxylic acids

Entry	Quinazolinone acid	Amine	Time (h)	Product	Yield (%)
1	6d	Benzylamine^a^	2	8a	95
2	6e	4-Methoxybenzylamine	24	8b	96
3	6f	4-Methoxybenzylamine	24	8c	98
4	6f	Benzylamine	24	8d	88
5	6d	2-Aminobenzimidazole	2	8e	—
6	6d	Isopropylamine	2	8f	91
7	6e	Benzylamine	4	8g	78
8	6e	Isobutylamine	8	8h	66
9	6f	2-Amino-6-nitrobenzothiazole	2	8i	—
10	6d	Isobutylamine	4	8j	85
11	6e	2-Aminobenzothiazole	8	8k	—
12	6d	4-Methoxyphenethylamine	2	8l	74
13	6f	4-Chloroaniline	24	8m	—
14	6e	Isopropylamine	24	8n	60
15	6d	4-Methoxybenzylamine	2	8o	92
16	6d	2-Ethylhexan-1-amine	2	8p	91
17	6e	2-(3,4-Dimethoxyphenyl)ethan-1-amine	2	8q	58
18	6d	2-(3,4-Dimethoxyphenyl)ethan-1-amine	2	8r	89
19	6d	2-(4-Chlorophenyl)ethan-1-amine	2	8 s	93
20	6f	2-(4-Chlorophenyl)ethan-1-amine	2	8 t	77

a A yield of 65% was obtained using Novozym 435 in acetone at 50 °C.

**Scheme 10:**
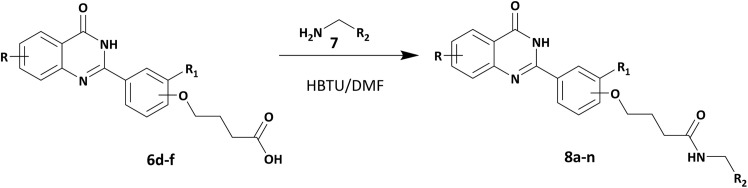
HBTU-catalysed direct amidation of quinazolinone carboxylic acids 6.

Given the limitations of the available biocatalysts, HBTU was employed as a chemical coupling agent, which afforded excellent results for the condensation of quinazolinone carboxylic acid 6 with aliphatic and benzyl amines 7 to afford the amide 8 (Table 6). Various substituted amines, including aliphatic and benzyl derivatives, were coupled with 2-substituted quinazolinone acids in DMF using HBTU and a base (*N*-methylmorpholine), affording high yields under mild conditions (Scheme 10).

However, this method was also not universally applicable, and no amide products were detected with aromatic amines (2-aminobenzimidazole, 2-aminobenzothiazole, 2-amino-6-nitrobenzothiazole) or 4-chloroaniline (Table 6, entries 5, 9, 11, and 13).

We acknowledge the current limited scope of the final lipase-catalysed biocatalytic step and the general issues associated with the alternative routes using stoichiometric coupling reagents (HBTU; EDC (1-ethyl-3-(3-dimethylaminopropyl)carbodiimide); thionyl chloride, HATU (hexafluorophosphate azabenzotriazole tetramethyl uronium); or CDI (1,1′-carbonyldiimidazole)), which generate large quantities of waste for amide synthesis.^78^ Amide syntheses in both small and large-scale processes still suffer from many problems.^79,80^ Despite these issues, this work provides a strong foundation for improving biocatalytic methods in multi-step organic synthesis.

## Conclusion

We have successfully developed an efficient chemoenzymatic method for the synthesis of novel 2-substituted quinazolin-4(3*H*)-ones incorporating linker and tail functionalities. This approach integrates biocatalytic transformations—nitrile hydratase-mediated hydrolysis, laccase-catalysed oxidative cyclisation, and lipase-mediated hydrolysis. Compared to conventional methods, this approach reduces reaction time and temperature while employing microwave irradiation and molecular oxygen (air) as a green and abundant oxidant. Amidation *via* lipase proved challenging, likely due to solubility constraints; future improvements may involve enzyme engineering to improve reaction outcomes. Overall, the method demonstrates the versatility of enzymes in the organic synthesis of quinazolinone and highlights their potential for greener and sustainable practices.

## Author contributions

Mudzuli Maphupha: conceptualisation, investigation, writing – original draft. Marushka Soobben: investigation, writing – draft. Charles B. de Koning: supervision – review & editing. Dean Brady: supervision, writing – review & editing.

## Conflicts of interest

There are no conflicts to declare.

## Supplementary Material

RA-OLF-D6RA02193J-s001

## Data Availability

All data supporting this study are available in the supplementary information (SI). Supplementary information: full experimental section, detailed synthesis of all intermediates, characterization data for all compounds (^1^H NMR, ^13^C NMR, FTIR, HRMS), along with aditional specture. See DOI: https://doi.org/10.1039/d6ra02193j.
