# Supplementary material for: A chemoenzymatic synthesis of amide-containing quinazolin-4(3H)-one derivatives
Source: RSC Adv. 2026 Jul 2;16(34):32311–20. doi: 10.1039/d6ra02193j (PMC13326023; doi:10.1039/d6ra02193j)
Supplement: RA-016-D6RA02193J-s001 [file RA-016-D6RA02193J-s001.pdf]

Supplementary Information (SI) for RSC Advances.
This journal is © The Royal Society of Chemistry 2026

SUPPORTING INFORMATION

A Chemoenzymatic Synthesis of Amide-Containing Quinazolin-4(*3H*)-one Derivatives

Mudzuli M. Maphupha[*a], Marushka Soobben[b], Charles De Koning[a], Dean Brady[a]

a. Molecular Sciences Institute, School of Chemistry, University of the Witwatersrand, PO Wits, 2050 Johannesburg, South Africa

b. Wits Industrial Biotechnology Consortium, School of Molecular and Cell Biology, Faculty of Science University of the Witwatersrand, Johannesburg 2050

Experimental

All reagents and solvents were commercially available and used as received without additional purification unless otherwise stated. Solvents were distilled before chromatographic separations were carried out. Thin-layer chromatography (TLC) was performed on Merck Aluminium foil-backed plates coated with silica gel (60 F254), with visualisation under UV light. Silica gel column chromatography was performed using Macherey-Nagel silica gel 60 (particle size 0.063 mm to 0.20 mm) purchased from Merck. Melting points (mp) were determined on a Stuart SMP10 instrument. Bruker AVANCE 300 MHz, Bruker AVANCE 400 MHz and Bruker AVANCE III 500 MHz spectrometers were used to record the ^{1}H and ^{13}C Nuclear Magnetic Resonance data in DMSO-d_6 or $CDCl_3$. Data processing of the spectra was performed using MestreNova Software under license from Mestrelab Research, CA, USA. Chemical shifts were expressed in ppm downfield against an internal standard, TMS (δ scale), and coupling constants were conveyed in Hertz.

High-resolution mass spectra were recorded on an inked Bruker Compact Q-TOF mass spectrometer (Bruker Daltonics, Bremen, Germany) using an ESI-positive source. A 10 µL volume of the sample was injected into the Dionex Ultimate 3000 UHPLC (Thermo Scientific, Dionex, Sunnyvale, California, USA) and run through a loop for one minute at 50% Solvent A consisting of 0.1 % formic acid in H_2O (v/v) and 50% solvent B composed of 0.1 % formic acid in acetonitrile (v/v) at a flow rate of 0.3 mL/min.

General procedure for the preparation of 2-aminobenzamide derives (2a-b)[1]

A wet nitrile hydratase active whole cell paste of *Rhodococcus rhodochrous* ATCC BAA-870 (50 mL) was added to a solution of 2-aminobenzonitrile derivatives (10.0 mmol) in 18 mL of Tris buffer (pH 7.6) and 10% acetone (v/v). The reaction mixture was stirred overnight at room temperature. The contents of the reaction mixture were extracted with ethyl acetate (30.0 mL) and washed with water (3 × 20.0 mL). The organic layer was separated and dried over $MgSO_4$, and the products were recrystallised from cold ethanol or purified by column chromatography (60% ethyl acetate/hexane) to afford the desired product **2a-b.**

2-aminobenzamide (2a)

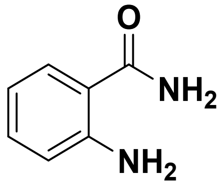

.

Stirring time = 24 h. Purified by column chromatography to afford a purple solid (1.27 g, 95%). R_f (40% EtOAc/hexane) 0.64. MP = 110 – 122 °C (lit. 109 – 110 ℃).[2] **^{1}H NMR (400**

MHz, DMSO-d_6) δ 7.73 (s, 1H), 7.53 (dd, *J* = 8.0, 1.6 Hz, 1H), 7.13 (ddd, *J* = 8.4, 7.0, 1.6 Hz, 1H), 7.07 (s, 1H), 6.68 (dd, *J* = 8.3, 1.2 Hz, 1H), 6.57 (s, 2H), 6.48 (ddd, *J* = 8.1, 7.0, 1.2 Hz, 1H). **^{13}C NMR (101 MHz, DMSO-d_6) δ** 171.7, 150.6, 132.3, 129.2, 116.8, 114.8, 114.1.

2-amino-5-nitrobenzamide (2b)

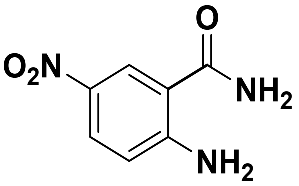

Stirring time = 24 h. Purified by recrystallisation to afford a yellow solid (0.833 g, 46%). R_f (60% EtOAc/hexane) 0.40. MP = 130 – 133 °C (lit. 128 – 134 ℃).[3] **^{1}H NMR (400 MHz, DMSO-d_6) δ** 8.56 (d, *J* = 2.7 Hz, 1H), 8.23 (s, 1H), 8.02 (dd, *J* = 9.2, 2.6 Hz, 1H), 7.97 – 7.85 (s, 2H), 7.42 (s, 1H), 6.80 (d, *J* = 9.3 Hz, 1H). **^{13}C NMR (101 MHz, DMSO-d_6) δ** 170.1, 156.1, 135.2, 127.9, 126.8, 116.4, 112.4.

General procedure for the synthesis of ether benzaldehydes 3(d-f)

Hydroxy benzaldehyde derivative (1 equivalent) was dissolved in anhydrous acetone and potassium carbonate (2 equivalents) was added; the contents were stirred at 50°C for 5 minutes under nitrogen gas. Ethyl 4-bromobutyrate (1 equivalent) was then introduced into the stirring solution. Finally, potassium iodide (1 equivalent) was added, and the reaction mixture was refluxed for 24 hours. Upon completion, the resultant mixture was concentrated, ethyl acetate (60.0 mL) was added, and the solution was washed with water (3 × 20.0 mL). The ethyl acetate layer was separated, treated with brine, and dried over $MgSO_4$. The crude mixture was purified by silica chromatography using hexane: ethyl acetate (9:1) to afford the desired ether hydroxy benzaldehyde derivatives **3(d-f)**.

Ethyl 4-(4-formylphenoxy)butanoate (3d)

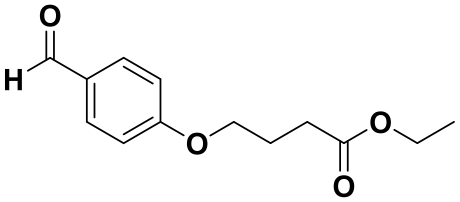

The crude product was purified by column chromatography eluting with 10% ethyl acetate in hexane, to obtain a pale-yellow oil containing a small amount of ethyl 4-bromobutyrate. R_f (60% EtOAc/hexane) 0.83. (lit.).[4] The product mixture was used in the subsequent reaction. **^{1}H NMR (400 MHz, $CDCl_3$) δ** 9.88 (s, 1H), 7.83 (d, J = 8.8 Hz, 2H),

6.99 (d, J = 8.7 Hz, 2H), 4.18 – 4.07 (m, 4H), 2.52 (t, 7.2 Hz, 2H), 2.19 – 2.11 (m, 2H), 1.30 – 1.23 (m, 3H). **^{13}C NMR (101 MHz, $CDCl_3$) δ** 190.7, 172.9, 163.8, 131.9, 129.94, 114.73, 67.12, 60.55, 30.59, 24.40, 14.21.

Ethyl 4-(3-formylphenoxy)butanoate (3e)

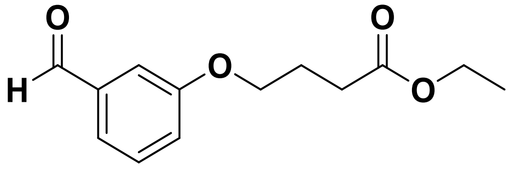

The crude product was purified by silica chromatography eluting with 10% ethyl acetate in hexane, to obtain a colourless oil (2.27 g, 96%). R_f (60% EtOAc/hexane) 0.83. (BP. lit. 175 – 185 ℃).[5] **^{1}H NMR (400 MHz, DMSO-d_6) δ** 9.91 (s, 1H), 7.49 – 7.42 (m, 2H), 7.35 (s, 1H), 7.22 – 7.19 (m, 1H), 4.02 – 3.97 (m, 4H), 2.41 (t, J = 7.3 Hz, 2H), 1.93 (p, J = 6.8 Hz, 2H), 1.11 (t, J = 7.1 Hz, 3H). **^{13}C NMR (101 MHz, DMSO-d_6) δ** 193.4, 172.9, 159.4, 138.0, 130.8, 130.1, 122.8, 114.0, 67.3, 60.3, 30.5, 24.5, 14.5.

Ethyl 4-(4-formyl-2-methoxyphenoxy)butanoate (3f)

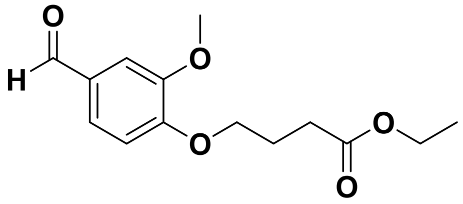

Purified by silica chromatography eluting with 10% ethyl acetate in hexane, to obtain a colourless oil (2.57 g, 97%). R_f (60% EtOAc/hexane) 0.84. (MP. lit. 75 – 76 ℃).[6] **^{1}H NMR (400 MHz, DMSO-d6) δ** 9.83 (s, 1H), 7.53 (dd, J = 8.2, 1.9 Hz, 1H), 7.39 (d, J = 1.9 Hz, 1H), 7.16 (d, J = 8.3 Hz, 1H), 4.12 – 4.03 (m, 4H), 3.84 (s, 3H), 2.47 (t, J = 7.3 Hz, 2H), 2.01 (p, J = 6.8 Hz, 2H), 1.18 (t, J = 7.1 Hz, 3H). **^{13}C NMR (101 MHz, DMSO-d6) δ** 191.7, 172.8, 153.8, 149.7, 130.1, 126.4, 112.5, 110.0, 67.9, 60.3, 55.9, 30.4, 24.5, 14.5.

General procedure for the preparation of 2-substituted quinazolinone derivatives (5)

A mixture of the 2-aminobenzamide derivative **2** (10 mmol) and benzaldehydes **3** (10 mmol) in a sealed microwave tube (neat). The mixture was then irradiated under microwave conditions (150 W, 130 °C) for 5 minutes while maintaining adequate stirring. After complete conversion of the starting materials (TLC) to aminal intermediate **4**, the contents were transferred to a 250 mL round-bottomed flask equipped with a magnetic stirrer bar by washing with DMSO (5 mL) to ensure quantitative transfer of all the solids. Additional DMSO (10 mL), laccase Novoprime Base 268 (0.50 g), and acetate buffer solution (0.1 M, pH 4.0, 5 mL) were added to the mixture. The mixture was then stirred at 70 °C with the flask open to air for the time given in **Table 2**. The crude product was precipitated out of the solution by the addition of water. Trituration of the crude product with ethanol afforded the quinazolinone product **5**.

2-phenylquinazolin-4(3*H*)-one (5a)

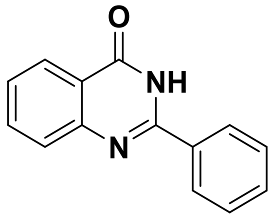

Trituration of the crude product with ethanol to obtain a white solid (2.57 g, 95%). R_f (60% EtOAc/hexane) 0.84. MP = 240 – 242 °C (lit. 241 – 242 ℃).[7] **^{1}H NMR (400 MHz, DMSO-d_6)** δ 8.20 (s, 1H), 8.17 (d, *J* = 8.8 Hz, 2H), 7.92 (d, *J* = 8.0 Hz, 2H), 7.70 – 7.64 (m, 1H), 7.61 (t, *J* = 7.5 Hz, 3H). **^{13}C NMR (101 MHz, DMSO-d_6)** δ 161.9, 154.6, 145.8, 135.5, 132.8, 130.8, 129.1, 129.0, 127.8, 126.6, 125.5, 120.9.

2-(3,4-dimethoxyphenyl)quinazolin-4(3*H*)-one (5b)

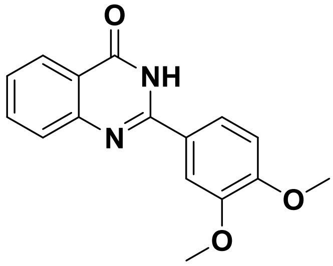

Trituration of the crude product with ethanol to obtain a white solid (2.78 g, 99%). R_f (60% EtOAc/hexane) 0.32. MP = 230 – 232 °C (lit. 222 – 224 ℃).[8] **^{1}H NMR (400 MHz, DMSO-d_6)** δ 12.42 (s, 1H), 8.14 (d, *J* = 7.6 Hz, 1H), 7.88 (d, *J* = 8.3 Hz, 1H), 7.82 (m, 2H), 7.72 (d, *J* = 7.8 Hz, 1H), 7.49 (t, *J* = 6.9 Hz, 1H), 7.12 (d, *J* = 8.1 Hz, 1H), 3.89 (s, 3H), 3.86 (s, 3H). **^{13}C NMR (101 MHz, DMSO-d_6)** δ 162.8, 152.3, 152.0, 149.2, 149.0, 134.9, 127.6, 126.5, 126.2, 125.2, 121.6, 121.1, 111.8, 111.1, 56.1.

2-(3,4,5-trimethoxyphenyl)quinazolin-4(*3H*)-one (5c)

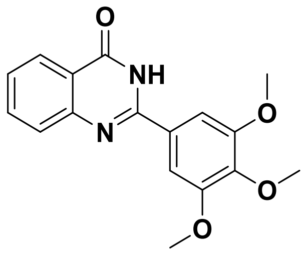

Trituration of the crude product with ethanol to obtain a white solid (2.78 g, 99%). R_f (60% EtOAc/hexane) 0.74. MP = 251 – 255 °C (lit. 258 – 260 ℃).[9] **^{1}H NMR (400 MHz, DMSO-d_6)** δ 12.51 (s, 1H), 8.15 (d, *J* = 7.8 Hz, 1H), 7.83 (t, *J* = 7.5 Hz, 1H), 7.75 (d, *J* = 8.0 Hz, 1H), 7.57 (s, 2H), 7.51 (t, *J* = 7.4 Hz, 1H), 3.91 (s, 6H), 3.76 (s, 3H). **^{13}C NMR (101 MHz, DMSO-d_6) δ** 162.7, 153.3, 152.1, 149.1, 140.7, 135.0, 128.1, 127.9, 126.9, 126.3, 121.2, 105.6, 60.6, 56.5.

Ethyl 4-(4-(4-oxo-3,4-dihydroquinazolin-2-yl)phenoxy)butanoate (5d)

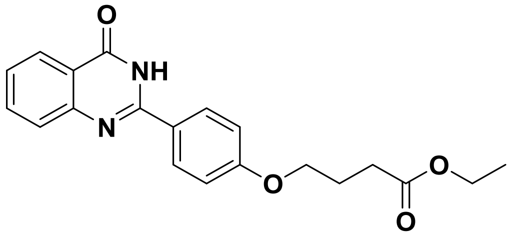

Trituration of the crude product with ethanol to obtain a cream-white solid (3.37 g, 96%). R_f (60% EtOAc/hexane) 0.73. MP = 180 – 184 °C. **IR (KBr) (cm^{-1})**: 3179, 2964, 1730, 1447, 1339. **^{1}H NMR (400 MHz, DMSO-*d*$_6$)** δ 12.37 (s, 1H), 8.18 (d, *J* = 8.8 Hz, 2H), 8.13 (d, *J* = 7.9 Hz, 1H), 7.81 (t, *J* = 8.3 Hz, 1H), 7.70 (d, *J* = 8.1 Hz, 1H), 7.48 (t, *J* = 7.5 Hz, 1H), 7.07 (d, *J* = 8.8 Hz, 2H), 4.08 (q, *J* = 7.3 Hz, 4H), 2.52 – 2.43 (m, 2H), 2.01 (p, *J* = 6.9 Hz, 2H), 1.19 (t, *J* = 7.1 Hz, 3H). **^{13}C NMR (101 MHz, DMSO-*d*$_6$)** δ 172.9, 162.8, 161.5, 152.37, 149.3, 134.9, 129.9, 126.5, 126.2, 125.2, 121.1, 114.8, 67.2, 60.3, 30.5, 24.5, 14.5. **HRMS** m/z calcd for $C_{20}H_{20}N_2O_4$: 352.1423, found $[M+H]^+$ 353.1478.

Ethyl 4-(3-(4-oxo-3,4-dihydroquinazolin-2-yl)phenoxy)butanoate (5e)

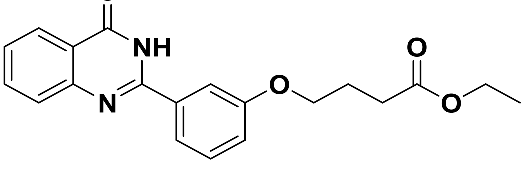

Trituration of the crude product with ethanol to obtain a cream-white solid (3.17 g, 90%). R_f (60% EtOAc/hexane) 0.75. MP = 154 – 160 °C. **IR (KBr) (cm^{-1})**: 3165, 2927, 1735, 1452, 1328. **^{1}H NMR (400 MHz, DMSO-*d*$_6$)** δ 12.54 (s, 1H), 8.16 (dd, *J* = 7.9, 1.2 Hz, 1H), 7.87 – 7.72 (m, 4H), 7.57 – 7.50 (m, 1H), 7.45 (t, *J* = 8.0 Hz, 1H), 7.14 (dd, *J* = 8.2, 1.8 Hz, 1H), 4.15 – 4.04 (m, 4H), 2.48 (m, 2H), 2.02 (p, *J* = 6.7 Hz, 2H), 1.19 (t, *J* = 7.1 Hz, 3H). **^{13}C NMR (101 MHz, DMSO-*d*$_6$)** δ 173.0, 162.7, 159.0, 149.1, 135.1, 134.4, 130.2, 127.9, 127.1,

126.3, 121.4, 120.6, 118.5, 113.5, 67.2, 60.3, 30.6, 24.6, 14.5. **HRMS** m/z calcd for $C_{20}H_{20}N_2O_4$: 352.1423, found $[M+H]^+$ 353.1479.

Ethyl 4-(2-methoxy-4-(6-nitro-4-oxo-3,4-dihydroquinazolin-2-yl)phenoxy) butanoate (5f)

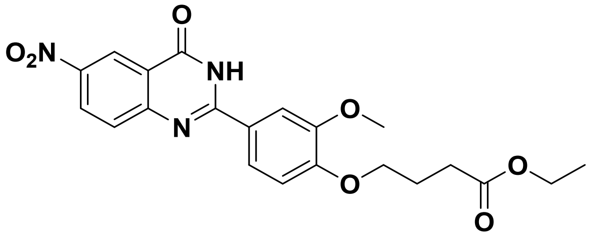

Trituration of the crude product with ethanol to obtain a yellow solid (4.21 g, 99%). R_f (60% EtOAc/hexane) 0.65. MP = 225 – 227 °C. **IR (KBr) (cm^{-1})**: 3115, 2905, 1665, 1472, 1332. **^{1}H NMR (400 MHz, DMSO) δ** 12.81 (s, 1H), 8.75 (d, J = 2.8 Hz, 1H), 8.47 (dd, J = 9.0, 2.8 Hz, 1H), 7.86 (dd, J = 8.5, 2.2 Hz, 1H), 7.82 (s, 1H), 7.80 (d, J = 2.0 Hz, 1H), 7.08 (d, J = 8.6 Hz, 1H),3.99-4.03 (m, 4H), 3.83 (s, 2H), 2.42 (t, *J* = 7.2 Hz, 2H) 1.95 (p, J = 6.9 Hz, 2H), 1.13 (t, J = 7.1 Hz, 3H). **^{13}C NMR (101 MHz, DMSO) δ** 172.9, 155.7, 151.9, 149.1, 144.6, 129.2, 128.8, 122.5, 122.4, 121.0, 112.8, 111.61, 67.8, 60.3, 56.2, 30.5, 24.6, 14.5. **HRMS** m/z calcd for $C_{21}H_{21}N_3O_7$: 427.1380, found $[M+H]^+$ 427.1430.

General procedure for the preparation of 2-substituted quinazolinone acids (6)

The following methods were used in the synthesis of quinazolinone acids **6d-f**.

Method A.

In a 100 mL round-bottom flask was added the quinazolinone ester **5d-f** (5 mmol) in a phosphate buffer (0.2M, pH 7.4) and 70% DMSO (v/v) (total volume of 10 mL). Then, 3Å molecular sieve (0.10 g) and immobilised CAL-B (0.10 g) were added to the mixture. The reaction mixture was heated to 30°C for 3 days. The product was precipitated out of the solution by the addition of water, and 3 M HCl (2 x 2 mL) was added to bring the pH of the solution to 3. Trituration of the crude product with ethanol afforded the quinazolinone acid **6d-f**.

Method B.

The same as **Method A**, except that DMF was used instead of DMSO.

Method C.

The same as **Method A**, except that Acetone was used instead of DMSO.

4-(4-(4-Oxo-3,4-dihydroquinazolin-2-yl)phenoxy)butanoic acid (6d)

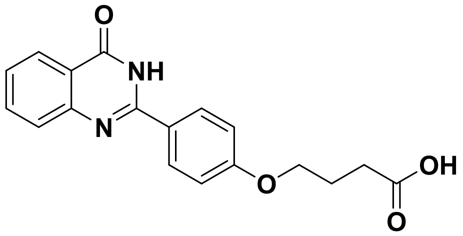

Method A

Trituration of the crude product with ethanol to obtain a cream-white solid (1.38 g, 86%). R_f (60% EtOAc/hexane) 0.29. MP = 220 – 224 °C. **IR (KBr) (cm^{-1})**: 3175, 2919, 1699, 1473, 1349. **^{1}H NMR (300 MHz, DMSO-*d*$_6$) δ** 12.40 (s, 1H), 12.19 (s, 1H), 8.18 (d, *J* = 8.9 Hz, 2H), 8.13 (dd, *J* = 7.9, 1.5 Hz, 1H), 7.81 (ddd, *J* = 8.5, 7.0, 1.6 Hz, 1H), 7.70 (dd, *J* = 8.3, 1.1 Hz, 1H), 7.48 (ddd, *J* = 8.1, 7.0, 1.3 Hz, 1H), 7.08 (d, *J* = 8.9 Hz, 2H), 4.09 (t, *J* = 6.5 Hz, 2H), 2.41 (t, *J* = 7.3 Hz, 2H), 1.98 (p, *J* = 6.9 Hz, 2H). **^{13}C NMR (75 MHz, DMSO-*d*$_6$) δ** 174.0, 162.2, 161.0, 151.8, 148.8, 134.5, 129.4, 127.2, 126.0, 125. 124. 120.6, 114.3, 66.8, 30.0, 24.1. **HRMS** m/z calcd for $C_{18}H_{16}N_2O_4$: 324.1110, found $[M+H]^+$ 325.1173.

Method B.

Stirring time = 24 h. Trituration of the crude product with ethanol to obtain a cream-white solid (1.33 g, 82 %).

Method C.

Stirring time = 5 days. Purified by column chromatography (80% ethyl acetate/hexane) to afford a cream-white solid (0.91 g, 56 %).

4-(3-(4-Oxo-3,4-dihydroquinazolin-2-yl)phenoxy)butanoic acid (6e)

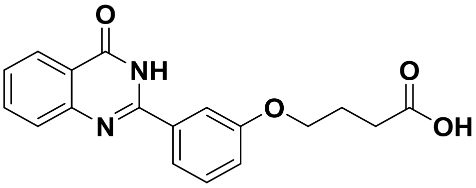

Method A.

Trituration of the crude product with ethanol to obtain a cream-white solid (1.06 g, 65%). R_f (60% EtOAc/hexane) 0.31. MP = 218 – 222 °C. **IR (KBr) (cm^{-1})**: 3125, 2889, 1700, 1462, 1322. **^{1}H NMR (400 MHz, DMSO-*d*$_6$) δ** 12.57 (s, 1H), 8.15 (d, *J* = 7.8 Hz, 1H), 7.84 (t, *J* = 7.4 Hz, 1H), 7.82 – 7.70 (m, 3H), 7.53 (t, *J* = 7.4 Hz, 1H), 7.45 (t, *J* = 8.0 Hz, 1H), 7.15 (dd, *J* = 8.2, 2.5 Hz, 1H), 4.10 (t, *J* = 6.4 Hz, 2H), 2.43 (t, *J* = 7.3 Hz, 2H), 1.99 (p, *J* = 6.6 Hz, 2H). **^{13}C NMR (101 MHz, DMSO-*d*$_6$) δ** 174.5, 162.7, 159.0, 152.5, 148.9, 135.0, 134.3, 130.2, 127.8, 127.1, 126.3, 121.4, 120.6, 118.5, 113.5, 67.3, 30.6, 24.7. **HRMS** m/z calcd for $C_{18}H_{16}N_2O_4$: 324.1110, found $[M+H]^+$ 325.1172.

Method B.

Stirring time = 72 h. Trituration of the crude product with ethanol to obtain a cream-white solid (1.24 g, 76 %).

Method C.

Stirring time = 5 days. Purified by column chromatography (80% ethyl acetate/hexane) to afford a cream-white solid (0.77 g, 48 %).

4-(2-Methoxy-4-(6-nitro-4-oxo-3,4-dihydroquinazolin-2-yl)phenoxy) butanoic acid (6f)

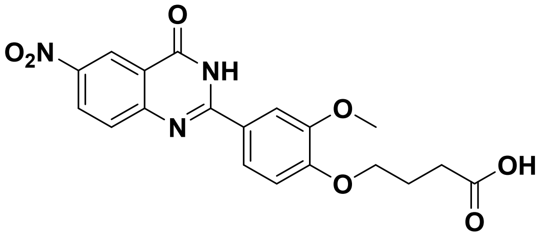

Method A.

Trituration of the crude product with ethanol to obtain yellow solid (1.39 g, 70%). R_f (60% EtOAc/hexane) 0.51. MP = 240 – 244 °C. **IR (KBr) (cm^{-1})**: 3162, 2991, 1712, 1558, 1325. **^{1}H NMR (400 MHz, DMSO-*d*$_6$) δ** 12.59 (s, 1H), 8.76 (d, *J* = 2.7 Hz, 1H), 8.48 (dd, *J* =

9.0, 2.8 Hz, 1H), 7.88 (dd, *J* = 8.5, 2.2 Hz, 1H), 7.82 (s, 1H), 7.80 (d, *J* = 6.4 Hz, 1H), 7.10 (d, *J* = 8.6 Hz, 1H), 4.07 (t, *J* = 6.4 Hz, 2H), 2.41 (t, *J* = 7.3 Hz, 2H), 1.98 (p, *J* = 7.0 Hz, 2H). **^{13}C NMR (101 MHz, DMSO-d_6)** δ 174.5, 162.3, 155.6, 153.6, 151.9, 149.0, 144.5, 129.1, 128.7, 124.4, 122.5, 122.4, 120.9, 112.7, 111.5, 67.8, 56.1, 30.4, 24.6. **HRMS** m/z calcd for $C_{19}H_{17}N_3O_7$: 399.1066, found [M+H]+ 400.1116.

Method B.

Stirring time = 24 h. Trituration of the crude product with ethanol to obtain a cream white solid (1.03 g, 52 %).

Method C.

Stirring time = 5 days. Purified by column chromatography (80% ethyl acetate/hexane) to afford a cream white solid (1.01 g, 51 %).

General procedure for the preparation of 2-substituted quinazolinone amides (8)

In a 100 mL round-bottom flask was successively introduced the amine **7** (0.35 mmol, 1 eq), and quinazolinone acid **6** (0.35 mmol, 1 eq) in DMF (5 mL). *N*-methylmorpholine (0.35 mmol, 1 eq) and 2-(1*H*-benzotriazol-1-yl)-1,1,3,3-tetramethyluronium hexafluorophosphate (HBTU)(0.35 mmol, 1 eq) are finally introduced. The reaction mixture is stirred at room temperature for 2h. The crude product was precipitated out of the solution by the addition of water into the mixture. Trituration of the crude product with acetone afforded the quinazolinone amide **8**.

***N*-benzyl-4-(4-(4-oxo-3,4-dihydroquinazolin-2-yl)phenoxy)butanamide (8a)**

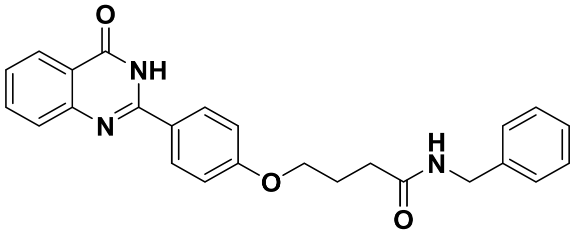

Trituration of the crude product with ethanol to obtain a white solid (137.4 mg, 95%). R_f (60% EtOAc/hexane) 0.28. MP = 244 – 247 °C. **IR (KBr) (cm^{-1})**: 3300, 3175, 2921, 1656, 1447, 1325. **^{1}H NMR (400 MHz, DMSO-*d*$_6$) δ** 12.25 (s, 1H), 8.45 (t, *J* = 6.0 Hz, 1H), 8.19 (d, *J* = 8.5 Hz, 2H), 8.14 (d, *J* = 8.0 Hz, 1H), 7.82 (t, *J* = 7.7 Hz, 1H), 7.71 (d, *J* = 8.2 Hz, 1H), 7.49 (t, *J* = 7.6 Hz, 1H), 7.30 (d, *J* = 7.1 Hz, 2H), 7.25 – 7.21 (m, 3H), 7.07 (d, *J* = 8.5 Hz, 2H), 4.29 (d, *J* = 6.0 Hz, 2H), 4.08 (t, *J* = 6.3 Hz, 2H), 2.36 (t, *J* = 7.4 Hz, 2H), 2.02 (q, *J* = 6.8 Hz, 2H). **^{13}C NMR (101 MHz, DMSO-*d*$_6$) δ** 171.9, 162.8, 161.6, 152.3, 149.3, 140.0, 135.0, 129.9, 128.7, 127.6, 127.1, 126.5, 126.2, 125.1, 121.1, 114.8, 67.6, 42.5, 32.0, 25.2. **HRMS** m/z calcd for $C_{25}H_{23}N_3O_3$: 413.1739; found $[M+H]^+$ 414.1839.

_N_-(4-methoxybenzyl)-4-(3-(4-oxo-3,4-dihydroquinazolin-2-yl)phenoxy) butanamide (8b)

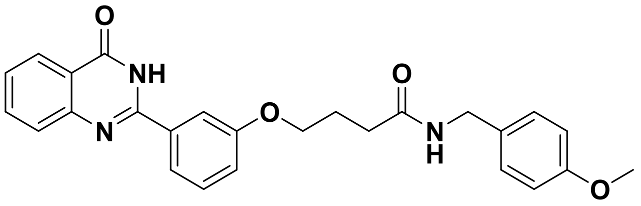

Trituration of the crude product with ethanol to obtain a white solid (148.9 mg, 95%). R_f (60% EtOAc/hexane) 0.56. MP = 225 – 229 °C. **IR (KBr) (cm^{-1})**: 3289, 3208, 2959, 1681, 1448, 1323. **^{1}H NMR (400 MHz, DMSO-*d*$_6$) δ** 12.52 (s, 1H), 8.36 (t, *J* = 5.9 Hz, 1H), 8.16 (d, *J* = 7.9 Hz, 1H), 7.88 – 7.71 (m, 4H), 7.53 (t, *J* = 7.5 Hz, 1H), 7.45 (t, *J* = 8.0 Hz, 1H), 7.16 (d, *J* = 8.5 Hz, 2H), 7.12 (s, 1H), 6.82 (d, *J* = 8.2 Hz, 2H), 4.21 (d, *J* = 5.8 Hz, 2H), 4.09 (t, *J* = 6.3 Hz, 2H), 3.67 (s, 3H), 2.35 (t, *J* = 7.3 Hz, 2H), 2.02 (p, *J* = 6.7 Hz, 1H). **^{13}C NMR (101 MHz, DMSO-*d*$_6$) δ** 171.87, 162.7, 159.1, 158.5, 152.5, 149.0, 135.0, 134.4, 132.0, 130.2, 128.9, 127.9, 127.0, 126.3, 121.4, 120.5, 118.5, 114.0, 113.5, 67.6, 55.4, 41.9, 32.1, 25.3. **HRMS** m/z calcd for $C_{26}H_{25}N_3O_4$: 443.1845; found $[M+H]^+$ 444.1845.

4-(2-Methoxy-4-(6-nitro-4-oxo-3,4-dihydroquinazolin-2-yl)phenoxy)-_N_-(4-methoxybenzyl)butanamide (8c)

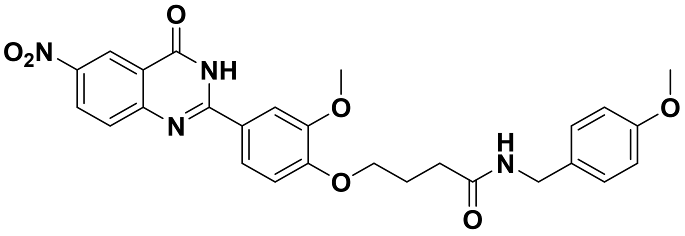

Trituration of the crude product with ethanol to obtain a yellow solid (177.9 mg, 98%). R_f (60% EtOAc/hexane) 0.58. MP = 252 – 256 °C. **IR (KBr) (cm^{-1})**: 3182, 3120, 2918, 1664, 1423, 1300. **^{1}H NMR (400 MHz, DMSO-*d$_6$*) δ** 12.89 (s, 1H), 8.82 (d, *J* = 2.8 Hz, 1H), 8.54 (dd, *J* = 9.0, 2.8 Hz, 1H), 8.36 (t, *J* = 5.9 Hz, 1H), 7.93 (dd, *J* = 8.5, 2.2 Hz, 1H), 7.88 (s, 1H), 7.86 (s, 1H), 7.17 (d, *J* = 8.6 Hz, 2H), 7.13 (d, *J* = 8.7 Hz, 1H), 6.86 (d, *J* = 8.7 Hz, 2H), 4.20 (d, *J* = 5.9 Hz, 2H), 4.08 (t, *J* = 6.4 Hz, 2H), 3.90 (s, 3H), 3.71 (s, 3H), 2.33 (t, *J* = 7.4 Hz, 2H), 2.00 (p, *J* = 6.8 Hz, 2H). **^{13}C NMR (101 MHz, DMSO-*d$_6$*)** δ 171.7, 162.3, 158.5, 155.7, 152.1, 149.1, 144.6, 132.0, 129.2, 128.9, 124.3, 122.5, 121.0, 114.1, 112.7, 111.5, 68.2, 56.2, 55.4, 32.0, 25.2. **HRMS** m/z calcd for $C_{27}H_{26}N_4O_7$: 518.1802; found $[M+H]^+$ 519.1892.

_N_-benzyl-4-(2-methoxy-4-(6-nitro-4-oxo-3,4-dihydroquinazolin-2-yl)phenoxy) butanamide (8d)

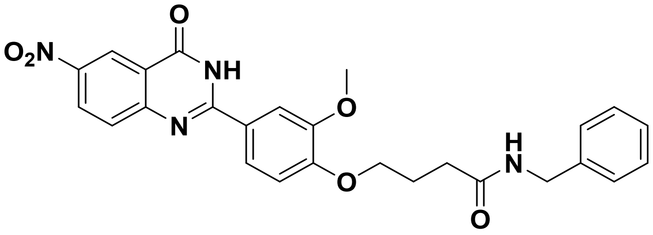

Trituration of the crude product with ethanol to obtain a yellow solid (177.9 mg, 98%). R_f (60% EtOAc/hexane) 0.58. MP = 262 – 266 °C. **IR (KBr) (cm^{-1})**: 3295, 3180, 2955, 1632, 1440, 1339. **^{1}H NMR (400 MHz, DMSO-*d$_6$*)** δ 12.85 (s, 1H), 8.79 (d, *J* = 2.7 Hz, 1H), 8.51 (dd, *J* = 9.0, 2.8 Hz, 1H), 8.44 (t, *J* = 6.0 Hz, 1H), 7.91 (dd, *J* = 8.6, 2.2 Hz, 1H), 7.86 (s, 1H), 7.85 – 7.83 (m, 1H), 7.34 – 7.29 (m, 2H), 7.25 (dt, *J* = 8.3, 2.2 Hz, 3H), 7.12 (d, *J* = 8.7 Hz, 1H), 4.29 (d, *J* = 5.9 Hz, 2H), 4.08 (t, *J* = 6.4 Hz, 2H), 3.89 (s, 3H), 2.36 (t, *J* = 7.4 Hz, 2H), 2.02 (p, *J* = 6.9 Hz, 2H). **^{13}C NMR (101 MHz, DMSO-d6) δ** 171.9, 162.7, 162.2, 155.6, 152.1, 149.1, 144.6, 140.0, 129.2, 128.8, 128.7, 127.6, 127.1, 124.3, 122.5, 120.9, 112.7, 111.5,

68.2, 56.1, 42.5, 32.02, 25.25. **HRMS** m/z calcd for $C_{26}H_{24}N_4O_6$: 488.1696; found $[M+H]^+$ 489.1793.

***N*-isopropyl-4-(4-(4-oxo-3,4-dihydroquinazolin-2-yl)phenoxy)butanamide (8f)**

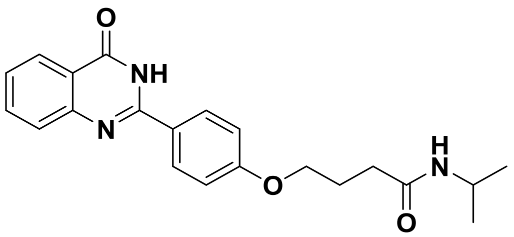

Trituration of the crude product with ethanol to obtain a white solid (116.4 mg, 91%). R_f (60% EtOAc/hexane) 0.18. MP = 272 – 274 °C. **IR (KBr) (cm^{-1})**: 3302, 3176, 2971, 1634, 1447, 1328. **^{1}H NMR (400 MHz, DMSO-*d_6*) δ** 12.42 (s, 1H), 8.18 (d, *J* = 8.9 Hz, 2H), 8.13 (dd, *J* = 8.0, 1.6 Hz, 1H), 7.84 – 7.76 (m, 2H), 7.71 (d, *J* = 7.9 Hz, 1H), 7.49 (ddd, *J* = 8.1, 7.0, 1.2 Hz, 1H), 7.08 (d, *J* = 9.0 Hz, 2H), 4.06 (t, *J* = 6.4 Hz, 2H), 3.84 (dq, *J* = 13.2, 6.6 Hz, 1H), 2.23 (t, *J* = 7.4 Hz, 2H), 1.95 (p, *J* = 6.8 Hz, 2H), 1.04 (d, *J* = 6.6 Hz, 6H). **^{13}C NMR (101 MHz, DMSO-d6)** δ 170.9, 162.8, 161.6, 152.5, 149.3, 135.0, 129.9, 127.7, 126.5, 126.2, 125.1, 121.1, 114.8, 67.7, 32.1, 25.2, 22.9. **HRMS** m/z calcd for $C_{21}H_{23}N_3O_3$: 365.1739; found $[M+H]^+$ 366.1837.

***N*-benzyl-4-(3-(4-oxo-3,4-dihydroquinazolin-2-yl)phenoxy)butanamide (8g)**

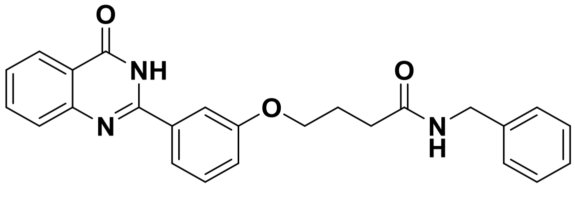

Trituration of the crude product with ethanol to obtain a white solid (112,6 mg, 78 %). R_f (60% EtOAc/hexane) 0.46. MP = 217– 219 °C. **IR (KBr) (cm^{-1})**: 3325, 3160, 2962, 1662, 1420, 1343.**^{1}H NMR (400 MHz, DMSO-*d_6*) δ** 11.42 (s, 1H), 8.44 (t, *J* = 5.8 Hz, 1H), 8.16 (dd, *J* = 7.9, 1.1 Hz, 1H), 7.85 (ddd, *J* = 8.4, 7.2, 1.5 Hz, 1H), 7.82 – 7.73 (m, 3H), 7.54 (ddd, J = 8.1, 7.1, 1.1 Hz, 1H), 7.46 (t, *J* = 8.0 Hz, 1H), 7.29 – 7.17 (m, 5H), 7.14 (dd, *J* = 8.2, 1.9 Hz, 1H), 4.29 (d, *J* = 5.9 Hz, 2H), 4.10 (t, *J* = 6.3 Hz, 2H), 2.38 (t, *J* = 7.3 Hz, 2H), 2.03 (p, *J* = 6.8 Hz, 2H). **^{13}C NMR (101 MHz, DMSO-*d_6*)** δ 172.0, 162.7, 159.1, 152.5, 140.0, 135.0, 134.4,

130.2, 128.7, 127.9, 127.6, 127.1, 127.1, 126.3, 121.4, 120.5, 118.5, 113.5, 67.6, 42.5, 32.1, 25.3. **HRMS** m/z calcd for $C_{25}H_{23}N_3O_3$: 413.1739; found $[M+H]^+$ 414.1837.

_N_-isobutyl-4-(3-(4-oxo-3,4-dihydroquinazolin-2-yl)phenoxy)butanamide (8h)

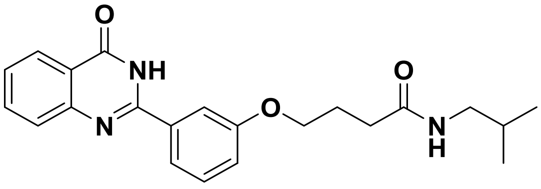

Trituration of the crude product with ethanol to obtain a white solid (87.6 mg, 66 %). R_f (60% EtOAc/hexane) 0.33. MP = 225 – 226 °C. **IR (KBr) (cm^{-1})**: 3280, 3117, 2971, 1654, 1427, 1329. **^{1}H NMR (400 MHz, DMSO-*d*$_6$) δ** 12.54 (s, 1H), 8.16 (dd, *J* = 8.0, 1.5 Hz, 1H), 7.90 (t, *J* = 5.8 Hz, 1H), 7.85 (ddd, *J* = 8.5, 7.1, 1.6 Hz, 1H), 7.81 – 7.72 (m, 3H), 7.53 (ddd, *J* = 8.1, 7.1, 1.2 Hz, 1H), 7.45 (t, *J* = 8.0 Hz, 1H), 7.14 (dd, *J* = 8.3, 1.7 Hz, 1H), 4.08 (t, *J* = 6.3 Hz, 2H), 2.88 (t, *J* = 6.3 Hz, 2H), 2.30 (t, *J* = 7.4 Hz, 2H), 1.98 (p, *J* = 6.5 Hz, 2H), 1.72 – 1.59 (m, *J* = 6.7 Hz, 1H), 0.82 (d, *J* = 6.7 Hz, 6H). **^{13}C NMR (101 MHz, DMSO-*d*$_6$) δ** 171.9, 159.1, 135.0, 134.4, 130.2, 127.0, 126.3, 121.4, 120.5, 118.5, 113.5, 67.7, 46.5, 32.1, 28.5, 25.4, 20.5. **HRMS** m/z calcd for $C_{22}H_{25}N_3O_3$: 379.1896; found $[M+H]^+$ 380.1990.

_N-i_sobutyl-4-(4-(4-oxo-3,4-dihydroquinazolin-2-yl)phenoxy)butanamide (8j)

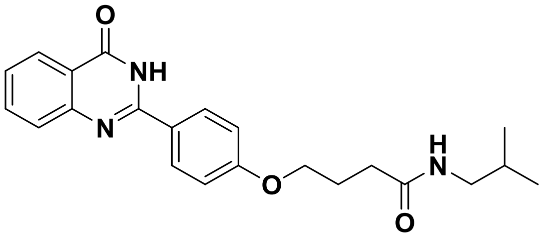

Trituration of the crude product with ethanol to obtain a white solid (112,9 mg, 85 %). R_f (60% EtOAc/hexane) 0.32. MP = 247 – 250 °C. **IR (KBr) (cm^{-1})**: 3304, 3175, 2965, 1669, 1447, 1325. **^{1}H NMR (400 MHz, DMSO-*d*$_6$) δ** 12.42 (s, 1H), 8.18 (d, *J* = 8.9 Hz, 2H), 8.13 (dd, *J* = 7.9, 1.6 Hz, 1H), 7.89 (t, *J* = 5.9 Hz, 1H), 7.82 (ddd, *J* = 8.5, 7.1, 1.6 Hz, 1H), 7.70 (d, *J* = 7.9 Hz, 1H), 7.49 (ddd, *J* = 8.1, 7.1, 1.2 Hz, 1H), 7.08 (d, *J* = 8.9 Hz, 2H), 4.06 (t, *J* = 6.4 Hz, 2H), 2.88 (t, *J* = 6.3 Hz, 2H), 2.28 (t, *J* = 7.4 Hz, 2H), 1.97 (p, *J* = 6.7 Hz, 2H), 1.73 – 1.60 (m, *J* = 6.6 Hz, 1H), 0.83 (d, *J* = 6.7 Hz, 6H). **^{13}C NMR (101 MHz, DMSO-d6) δ** 171.8, 162.7,

152.3, 135.0, 129.9, 127.7, 126.5, 126.2, 125.1, 121.1, 114.8, 67.7, 46.4, 32.0, 28.5, 25.3, 20.5. **HRMS** m/z calcd for $C_{22}H_{25}N_3O_3$: 379.1896; found $[M+H]^+$ 380.1995.

***N*-(4-methoxyphenethyl)-4-(4-(4-oxo-3,4-dihydroquinazolin-2-yl)phenoxy) butanamide (8l)**

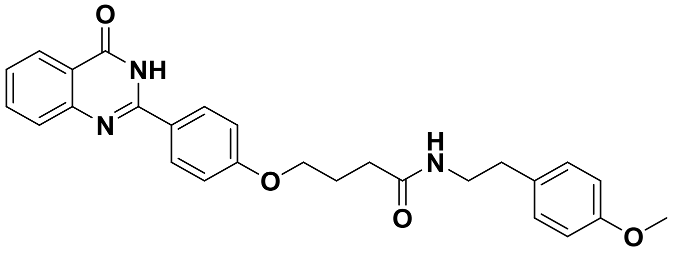

Trituration of the crude product with ethanol to obtain a white solid (118,2 mg, 74 %). R_f (60% EtOAc/hexane) 0.22. MP = 246 – 249 °C. **IR (KBr) (cm^{-1})**: 3305, 3175, 2931, 1639, 1447, 1326. **^{1}H NMR (400 MHz, DMSO-*d*$_6$) δ** 12.43 (s, 1H), 8.19 (d, *J* = 8.8 Hz, 2H), 8.14 (d, *J* = 7.7 Hz, 1H), 7.96 (t, *J* = 5.5 Hz, 1H), 7.82 (t, *J* = 7.2 Hz, 1H), 7.71 (d, *J* = 8.1 Hz, 1H), 7.49 (t, *J* = 7.4 Hz, 1H), 7.09 (dd, *J* = 13.8, 8.5 Hz, 4H), 6.82 (d, *J* = 8.5 Hz, 2H), 4.03 (t, *J* = 6.3 Hz, 2H), 3.69 (s, 3H), 3.24 (q, *J* = 6.8 Hz, 2H), 2.64 (t, *J* = 7.3 Hz, 2H), 2.25 (t, *J* = 7.3 Hz, 2H), 1.96 (p, *J* = 6.5 Hz, 2H). **^{13}C NMR (101 MHz, DMSO-*d*$_6$) δ** 171.8, 162.7, 161.6, 158.0, 152.3, 149.3, 135.0, 131.8, 130.0, 129.9, 127.7, 126.5, 126.2, 125.1, 121.1. 114.8, 114.1, 67.6, 55.3, 34.7, 32.0, 25.2. **HRMS** m/z calcd for $C_{27}H_{27}N_3O_4$: 457.2002; found $[M+H]^+$ 458.2096.

***N*-isopropyl-4-(3-(4-oxo-3,4-dihydroquinazolin-2-yl)phenoxy)butanamide (8n)**

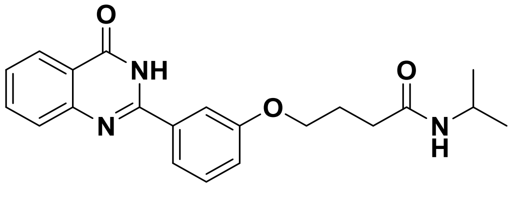

Trituration of the crude product with ethanol to obtain a white solid (78.6 mg, 60 %). R_f (60% EtOAc/hexane) 0.22. MP = 245 – 247 °C. **IR (KBr) (cm^{-1})**: 3307, 3143, 2964, 1638, 1448, 1344. **^{1}H NMR (400 MHz, DMSO-*d*$_6$) δ** 12.55 (s, 1H), 8.16 (d, *J* = 7.8 Hz, 1H), 7.85

(ddd, *J* = 8.5, 7.1, 1.6 Hz, 1H), 7.81 – 7.70 (m, 4H), 7.53 (t, *J* = 7.2 Hz, 1H), 7.45 (t, *J* = 8.0 Hz, 1H), 7.14 (dd, *J* = 8.1, 2.5 Hz, 1H), 4.08 (t, *J* = 6.3 Hz, 2H), 3.92 – 3.78 (m, *J* = 6.8 Hz, 1H), 2.24 (t, *J* = 7.4 Hz, 2H), 1.98 (q, *J* = 7.1, 6.4 Hz, 2H), 1.04 (d, *J* = 6.5 Hz, 6H). **^{13}C NMR (101 MHz, DMSO-d_6) δ** 170.9, 162.6, 159.1, 152.4, 149.1, 135.0, 134.4, 130.2, 127.9, 127.1, 126.3, 121.4, 120.5, 118.5, 113.5, 67.7, 32.2, 25.3, 22.9. **HRMS** m/z calcd for $C_{21}H_{23}N_3O_3$: 365.1739; found $[M+H]^+$ 366.1841.

_N_-(4-methoxybenzyl)-4-(4-(4-oxo-3,4-dihydroquinazolin-2-yl)phenoxy) butanamide (8o)

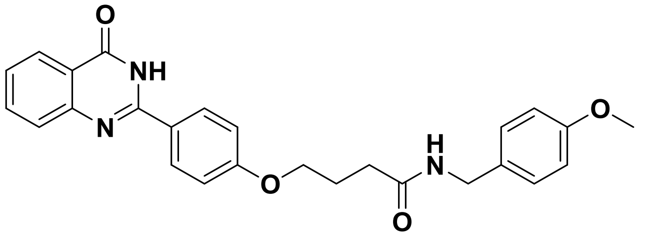

Trituration of the crude product with ethanol to obtain a white solid (142.8 mg, 92 %). R_f (60% EtOAc/hexane) 0.27. MP = 228 – 233 °C. **IR (KBr) (cm^{-1})**: 3379, 3185, 2967, 1666, 1472, 1345. **^{1}H NMR (400 MHz, DMSO-d_6) δ** 12.43 (s, 1H), 8.36 (t, *J* = 5.9 Hz, 1H), 8.19 (d, *J* = 9.0 Hz, 2H), 8.14 (dd, *J* = 8.0, 1.5 Hz, 1H), 7.82 (ddd, *J* = 8.5, 7.1, 1.6 Hz, 1H), 7.71 (d, *J* = 7.7 Hz, 1H), 7.49 (ddd, *J* = 8.1, 7.1, 1.2 Hz, 1H), 7.16 (d, *J* = 8.6 Hz, 2H), 7.06 (d, *J* = 9.0 Hz, 2H), 6.85 (d, *J* = 8.7 Hz, 2H), 4.21 (d, *J* = 5.9 Hz, 2H), 4.07 (t, *J* = 6.4 Hz, 2H), 3.70 (s, 3H), 2.33 (t, *J* = 7.3 Hz, 2H), 2.00 (p, *J* = 6.7 Hz, 1H). **^{13}C NMR (101 MHz, DMSO-d_6) δ** 171.8, 162.7, 161.6, 158.5, 152.3, 149.3, 135.0, 132.0, 129.9, 128.9, 127.7, 126.5, 126.2, 125.1, 121.1, 114.8, 114.1, 67.6, 55.4, 41.9, 32.0, 25.2. **HRMS** m/z calcd for $C_{26}H_{25}N_3O_4$: 443.1845; found $[M+H]^+$ 444.1954.

_N_-(2-ethylhexyl)-4-(4-(4-oxo-3,4-dihydroquinazolin-2-yl)phenoxy) butanamide (8p)

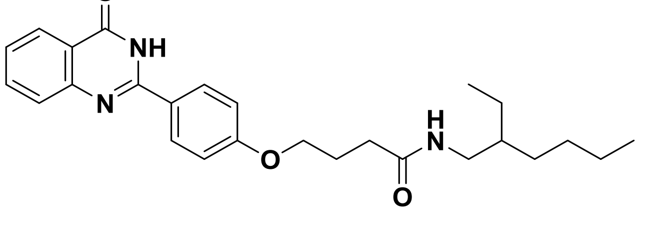

Trituration of the crude product with ethanol to obtain a white solid (138.6 mg, 91 %). R_f (60% EtOAc/hexane) 0.36. MP = 180 – 182 °C. **IR (KBr) (cm^{-1})**: 3305, 3173, 2957, 1652, 1447, 1345. **^{1}H NMR (400 MHz, DMSO-*d*$_6$) δ** 12.43 (s, 1H), 8.19 (d, *J* = 8.9 Hz, 2H), 8.13 (dd, *J* = 7.9, 1.6 Hz, 1H), 7.86 – 7.77 (m, 2H), 7.70 (d, *J* = 7.2 Hz, 1H), 7.49 (ddd, *J* = 8.1, 7.0, 1.1 Hz, 1H), 7.07 (d, *J* = 8.9 Hz, 2H), 4.06 (t, *J* = 6.4 Hz, 2H), 2.99 (t, *J* = 5.5 Hz, 2H), 2.28 (t, *J* = 7.3 Hz, 2H), 1.97 (p, *J* = 6.8 Hz, 2H), 1.38 – 1.26 (m, 1H), 1.31 – 1.16 (m, 8H), 0.87 – 0.76 (m, 6H). **^{13}C NMR (101 MHz, DMSO-*d*$_6$) δ** 171.9, 161.6, 152.3, 140.0, 135.0, 129.9, 128.7, 127.6, 127.1, 126.5, 126.2, 125.1, 121.1, 114.8, 67.6, 42.5, 32.0, 25.2. **HRMS** m/z calcd for $C_{26}H_{33}N_3O_3$: 435.2522; found $[M+H]^+$ 436.2614.

***N*-(3,4-dimethoxyphenethyl)-4-(3-(4-oxo-3,4-dihydroquinazolin-2-yl)phenoxy) butanamide (8q)**

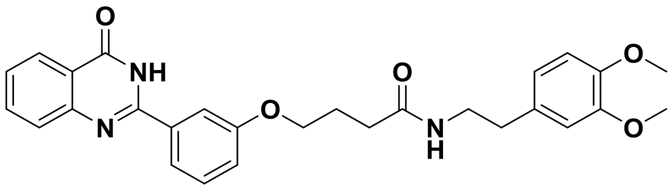

Trituration of the crude product with ethanol to obtain a white solid (98.7 mg, 58 %). R_f (60% EtOAc/hexane) 0.34. MP = 202 – 208 °C. **IR (KBr) (cm^{-1})**: 3249, 3149, 2981, 1623, 1457, 1333. **^{1}H NMR (400 MHz, DMSO-*d*$_6$) δ** 12.60 (s, 1H), 8.22 (d, *J* = 7.7 Hz, 1H), 8.04 (t, *J* = 5.6 Hz, 1H), 7.91 (t, *J* = 7.4 Hz, 1H), 7.85 (d, *J* = 7.8 Hz, 1H), 7.81 (d, *J* = 6.8 Hz, 2H), 7.59 (t, *J* = 7.4 Hz, 1H), 7.52 (t, *J* = 7.9 Hz, 1H), 7.20 (d, *J* = 7.9 Hz, 1H), 6.86 (d, *J* = 8.0 Hz, 2H), 6.74 (d, *J* = 7.9 Hz, 1H), 4.12 (t, *J* = 5.9 Hz, 2H), 3.78 (s, 3H), 3.73 (s, 3H), 3.35 – 3.29 (m, 2H), 2.70 (t, *J* = 7.0 Hz, 2H), 2.33 (t, *J* = 7.1 Hz, 2H), 2.03 (p, *J* = 7.0 Hz, 2H). **^{13}C NMR (101 MHz, DMSO-*d*$_6$) δ** 171.9, 159.1, 149.0, 147.6, 135.0, 134.4, 132.4, 130.2, 127.1, 126.3, 121.4, 120.8, 120.5, 118.5, 113.5, 112.9, 112.2, 67.6, 55.9, 55.7, 35.2, 32.1, 25.3. **HRMS** m/z calcd for $C_{28}H_{29}N_3O_5$: 487.2107; found $[M+H]^+$ 488.2143.

***N*-(3,4-dimethoxyphenethyl)-4-(4-(4-oxo-3,4-dihydroquinazolin-2-yl)phenoxy) butanamide (8r)**

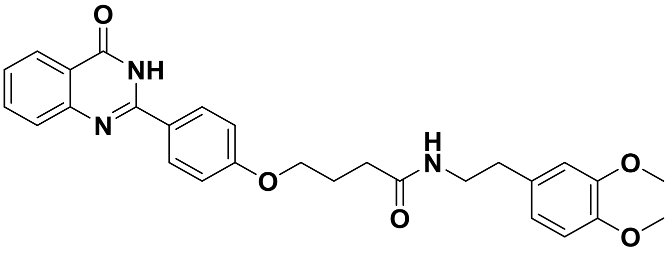

Trituration of the crude product with ethanol to obtain a white solid (151.6 mg, 89 %). R_f (60% EtOAc/hexane) 0.36. MP = 225 – 230 °C. **IR (KBr) (cm^{-1})**: 3330, 3075, 2962, 1640, 1441, 1322. **^{1}H NMR (400 MHz, DMSO-d6) δ** 12.34 (s, 1H), 8.11 (d, J = 8.9 Hz, 2H), 8.07 (dd, J = 7.9, 1.6 Hz, 1H), 7.90 (t, J = 5.6 Hz, 1H), 7.76 (ddd, J = 8.2, 7.0, 1.4 Hz, 1H), 7.64 (d, J = 7.9 Hz, 1H), 7.43 (ddd, J = 8.1, 7.1, 1.2 Hz, 1H), 7.00 (d, J = 8.9 Hz, 2H), 6.76 – 6.72 (m, 2H), 6.62 (dd, J = 8.1, 2.0 Hz, 1H), 3.97 (t, J = 6.3 Hz, 2H), 3.66 (s, 3H), 3.62 (s, 3H), 3.20 (q, J = 6.8 Hz, 2H), 2.57 (t, J = 7.3 Hz, 2H), 2.19 (t, J = 7.3 Hz, 2H), 1.89 (p, J = 6.8 Hz, 2H). **^{13}C NMR (101 MHz, DMSO-d_6) δ** 171.9, 162.7, 161.6, 152.3, 149.3, 148.9, 147.6, 135.0, 132.3, 129.9, 127.7, 126.6, 126.2, 125.1, 120.9, 114.8, 112.9, 112.2, 67.6, 55.9, 55.8, 35.1, 32.0, 25.2. **HRMS** m/z calcd for $C_{28}H_{29}N_3O_5$: 487.2107; found $[M+H]^+$ 488.2154.

_N_-(4-chlorophenethyl)-4-(4-(4-oxo-3,4-dihydroquinazolin-2-yl)phenoxy) butanamide (8s)

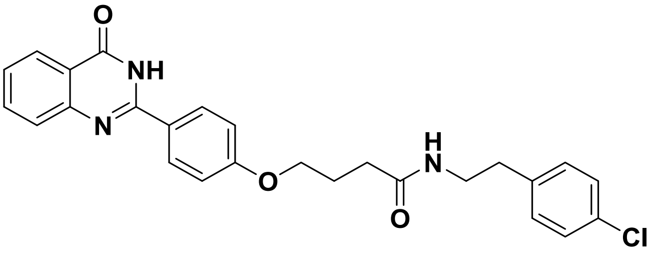

Trituration of the crude product with ethanol to obtain a white solid (150.1 mg, 93 %). R_f (60% EtOAc/hexane) 0.26. MP = 273 – 277 °C. **IR (KBr) (cm^{-1})**: 3329, 3087, 2962, 1644, 1455, 1344. **^{1}H NMR (400 MHz, DMSO-*d*$_6$) δ** 12.35 (s, 1H), 8.12 (d, *J* = 8.9 Hz, 2H), 8.07 (dd, *J* = 7.9, 1.6 Hz, 1H), 7.90 (t, *J* = 5.6 Hz, 1H), 7.75 (ddd, *J* = 8.5, 7.1, 1.6 Hz, 1H), 7.66 – 7.62 (m, 1H), 7.42 (ddd, *J* = 8.1, 7.0, 1.2 Hz, 1H), 7.25 (d, *J* = 8.4 Hz, 2H), 7.15 (d, *J* = 8.4 Hz, 2H), 7.00 (d, *J* = 8.9 Hz, 2H), 3.96 (t, *J* = 6.4 Hz, 2H), 3.21 (q, *J* = 6.8 Hz, 2H), 2.63 (t, *J* = 7.1 Hz, 2H), 2.17 (t, *J* = 7.3 Hz, 2H), 1.87 (p, *J* = 6.6 Hz, 2H). **^{13}C NMR (101 MHz, DMSO-*d*$_6$) δ** 171.8, 162.7, 161.6, 152.3, 149.4, 139.0, 135.0, 131.1, 131.0, 129.9, 128.6, 127.7, 126.5, 126.2, 125.1, 121.1, 114.8, 67.6, 34.8, 32.0, 25.2. **HRMS** m/z calcd for $C_{26}H_{24}ClN_3O_3$: 461.1506; found $[M+H]^+$ 462.1548.

***N*-(4-chlorophenethyl)-4-(2-methoxy-4-(6-nitro-4-oxo-3,4-dihydroquinazolin-2-yl)phenoxy)butanamide (8t)**

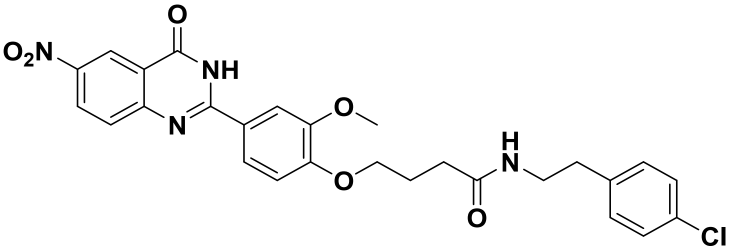

Trituration of the crude product with ethanol to obtain a white solid (144.4 mg, 77 %). R_f (60% EtOAc/hexane) 0.26. MP = 274 – 278 °C. **IR (KBr) (cm^{-1})**: 3379, 3175, 2967, 1666, 1412, 1345. **^{1}H NMR (400 MHz, DMSO) δ** 12.80 (s, 1H), 8.75 (s, 1H), 8.46 (d, J = 9.1 Hz, 1H), 7.94 – 7.83 (m, 2H), 7.80 (d, J = 8.6 Hz, 2H), 7.25 (d, J = 8.0 Hz, 2H), 7.15 (d, J = 8.0 Hz, 2H), 7.05 (d, J = 8.6 Hz, 1H), 3.96 (t, J = 6.5 Hz, 2H), 3.83 (s, 3H), 3.20 (t, J = 6.6 Hz, 3H), 2.64 (t, J = 7.3 Hz, 2H), 2.17 (t, J = 7.4 Hz, 2H), 1.88 (p, J = 8.0, 6.9 Hz, 2H). **^{13}C NMR (101 MHz, DMSO) δ** 171.8, 149.0, 144.5, 139.0, 131.0, 128.6, 122.6, 122.5, 112.7, 111.6, 68.2, 56.1, 34.8, 32.04, 25.2. **MS** m/z calcd for $C_{27}H_{25}ClN_4O_6$: 536.1463; found $[M+H]^+$ 537.1487.

References

1 M. Soobben, *University of the Witwatersrand (Masters dissertation)*, 2020, 57–59.

2 S. Hao, J. Yang, P. Liu, J. Xu, C. Yang and F. Li, *Org. Lett.*, 2021, **23**, 2553–2558.

3 N. D. Kushwaha, S. J. Zamisa, B. Kushwaha, A. Sharma, F. Kayamba, S. R. Merugu, A. M. Ganai, V. A. Obakachi, F. Albericio and R. Karpoormath, *J. Mol. Struct.*, 2021, **1231**, 129951.

4 W. Zhu, Y. H. Gao, P. Y. Liao, D. Y. Chen, N. N. Sun, P. A. Nguyen Thi, Y. J. Yan, X. F. Wu and Z. L. Chen, *Eur. J. Med. Chem.*, 2018, **160**, 146–156.

5 D. G. Cooper and G. S. Sach, US4524071, 1983.

6 K. Wada, J. Y. Lee, H. Y. Hung, Q. Shi, L. Lin, Y. Zhao, M. Goto, P. C. Yang, S. C. Kuo, H. W. Chen and K. H. Lee, *Bioorg. Med. Chem.*, 2015, **23**, 1507–1514.

7 M. Chi, W. L. Xiong, D. Z. Yang, C. Bin Fan, R. W. Shi, S. S. Gong and Q. Sun, *J. Phys. Org. Chem.*, 2022, **4**, 1–9.

8 R. Gupta, G. Arora, P. Yadav, R. Dixit, A. Srivastava and R. K. Sharma, *Dalton Transactions*, 2021, **50**, 890–898.

9 S. L. Matcha, B. K. Karasala, S. M. Botsa and S. Vidavalur, *J. Heterocycl. Chem.*, 2021, **10**, 1955–1961.

Spectroscopic Data

Ethyl 4-(4-(4-oxo-3,4-dihydroquinazolin-2-yl)phenoxy)butanoate (5d)

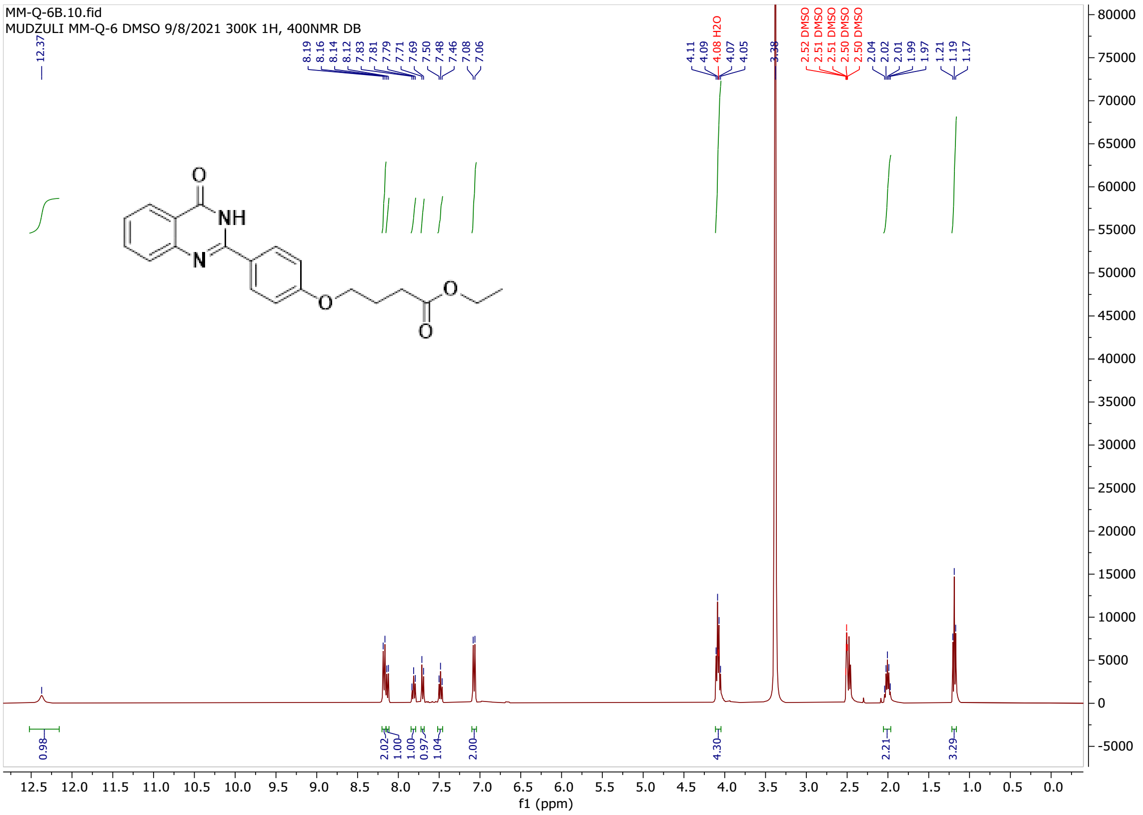

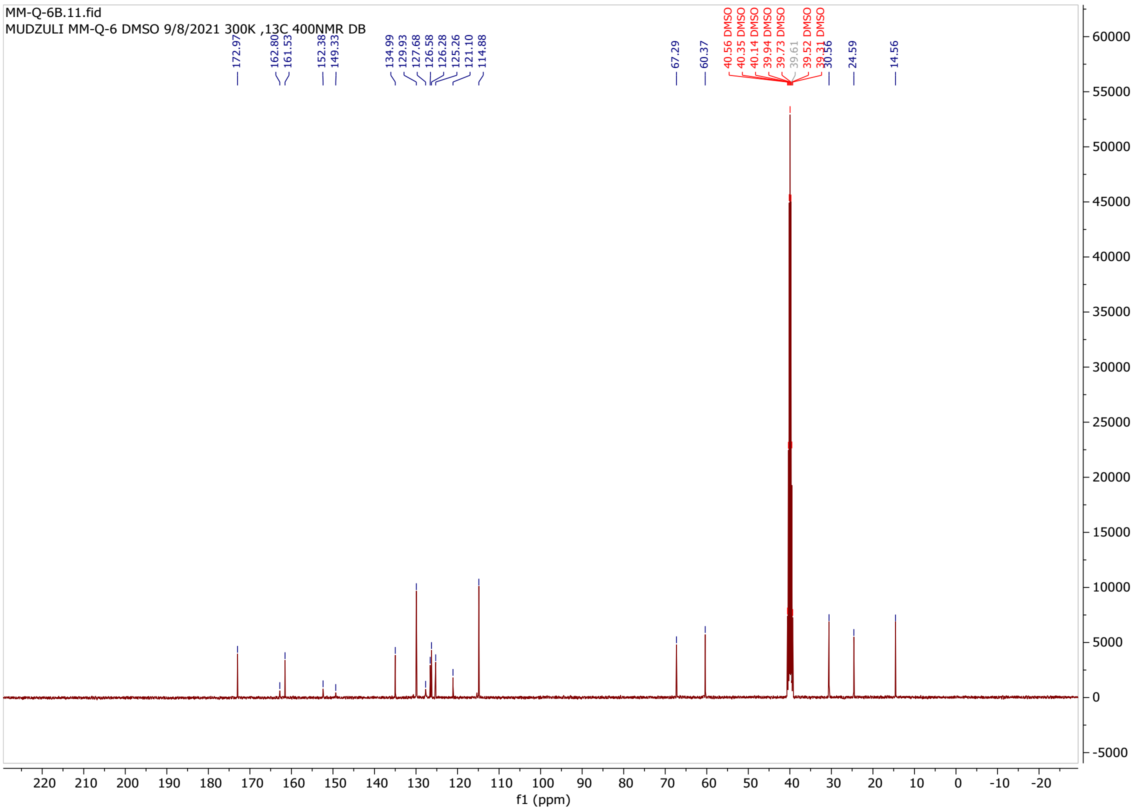

Ethyl 4-(3-(4-oxo-3,4-dihydroquinazolin-2-yl)phenoxy)butanoate (5e)

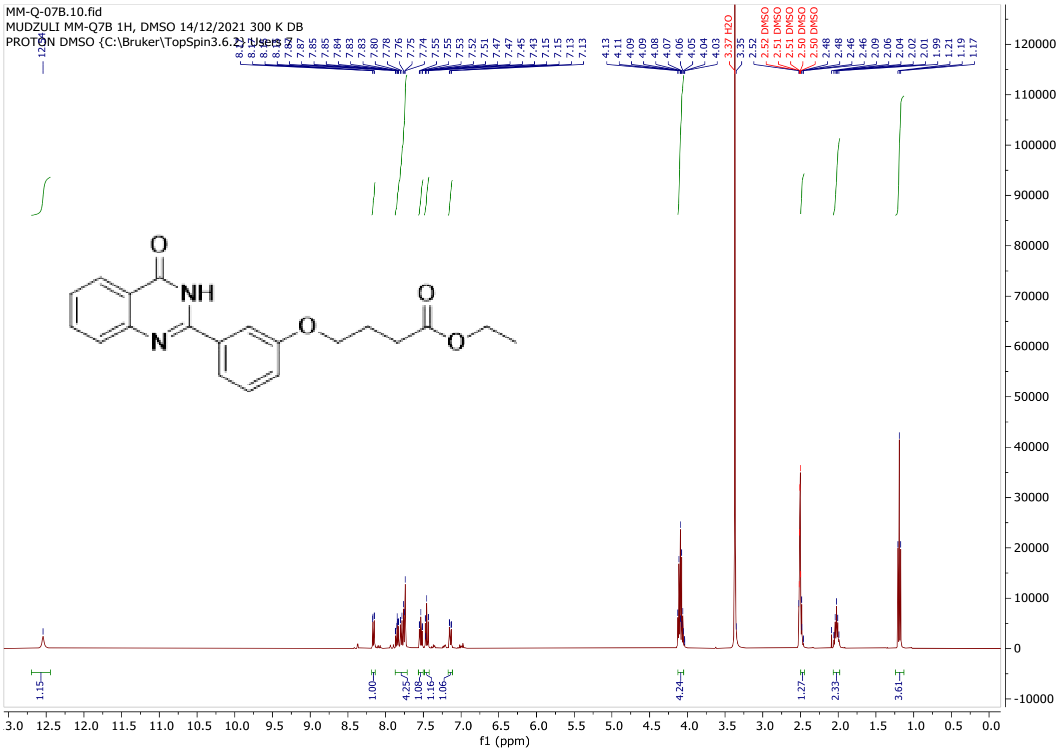

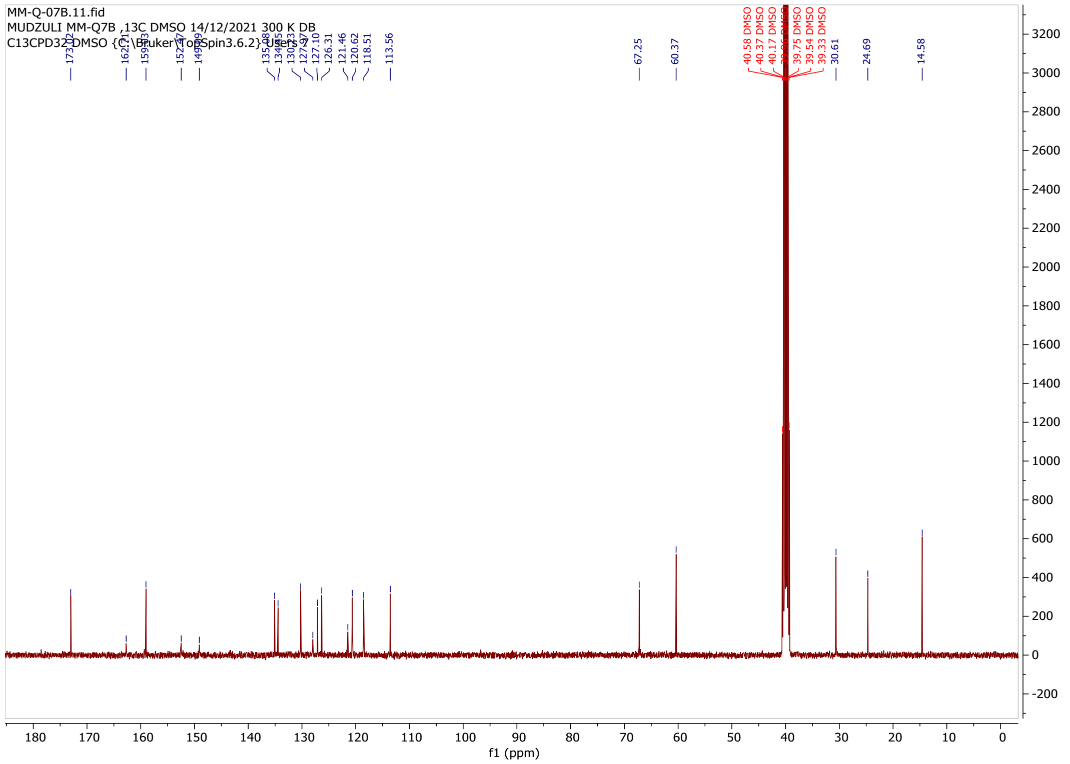

4-(4-(4-Oxo-3,4-dihydroquinazolin-2-yl)phenoxy)butanoic acid (6d)

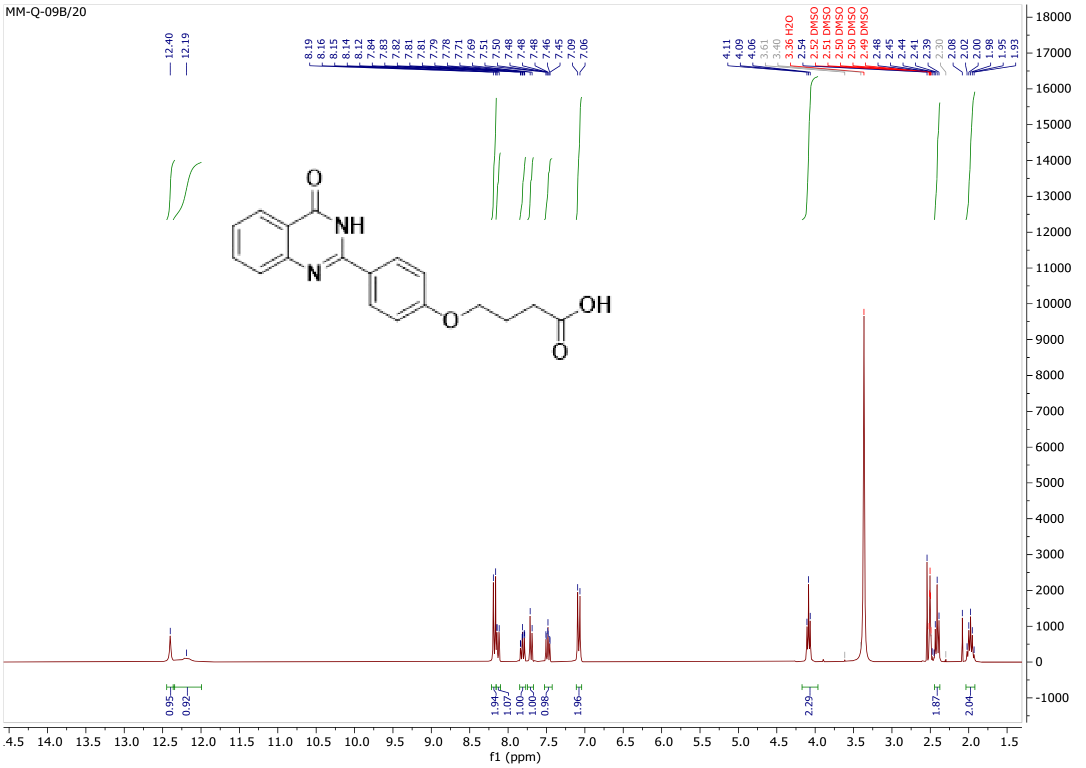
MM-Q-09B/20
12.40
12.19
8.19 8.16 8.15 8.14 8.12 7.84 7.83 7.82 7.81 7.81 7.79 7.78 7.71 7.69 7.51 7.50 7.48 7.48 7.48 7.46 7.45 7.09 7.06
4.11 4.09 4.06 3.61 3.40
3.36 H2O
2.54
2.52 DMSO 2.51 DMSO 2.50 DMSO 2.50 DMSO 2.49 DMSO
2.48 2.45 2.44 2.41 2.39 2.30 2.08 2.02 2.00 1.98 1.95 1.93
O
NH
N
O
OH
O
0.95
0.92
1.94
1.07
1.00
1.00
0.98
1.96
2.29
1.87
2.04
f1 (ppm)

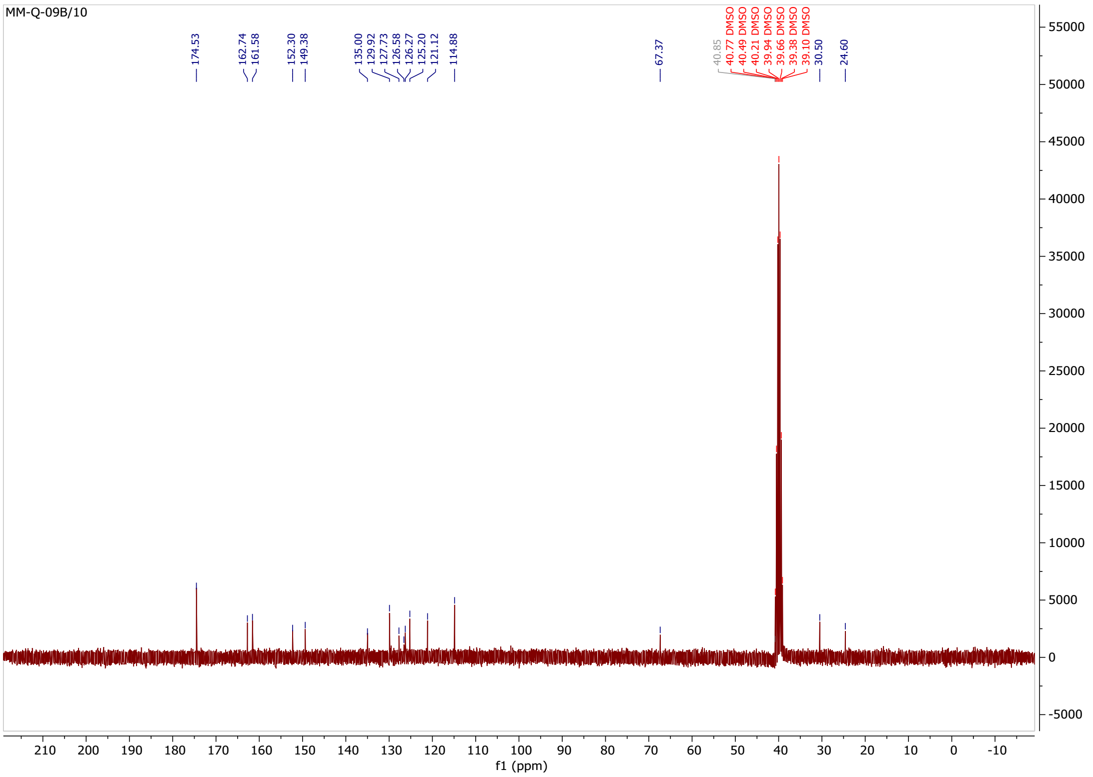
MM-Q-09B/10
174.53
162.74 161.58
152.30 149.38
135.00 129.92 127.73 126.58 126.27 125.20 121.12
114.88
67.37
40.85
40.77 DMSO 40.49 DMSO 40.21 DMSO 39.94 DMSO 39.66 DMSO 39.38 DMSO 39.10 DMSO
30.50
24.60
f1 (ppm)

4-(3-(4-Oxo-3,4-dihydroquinazolin-2-yl)phenoxy)butanoic acid (6e)

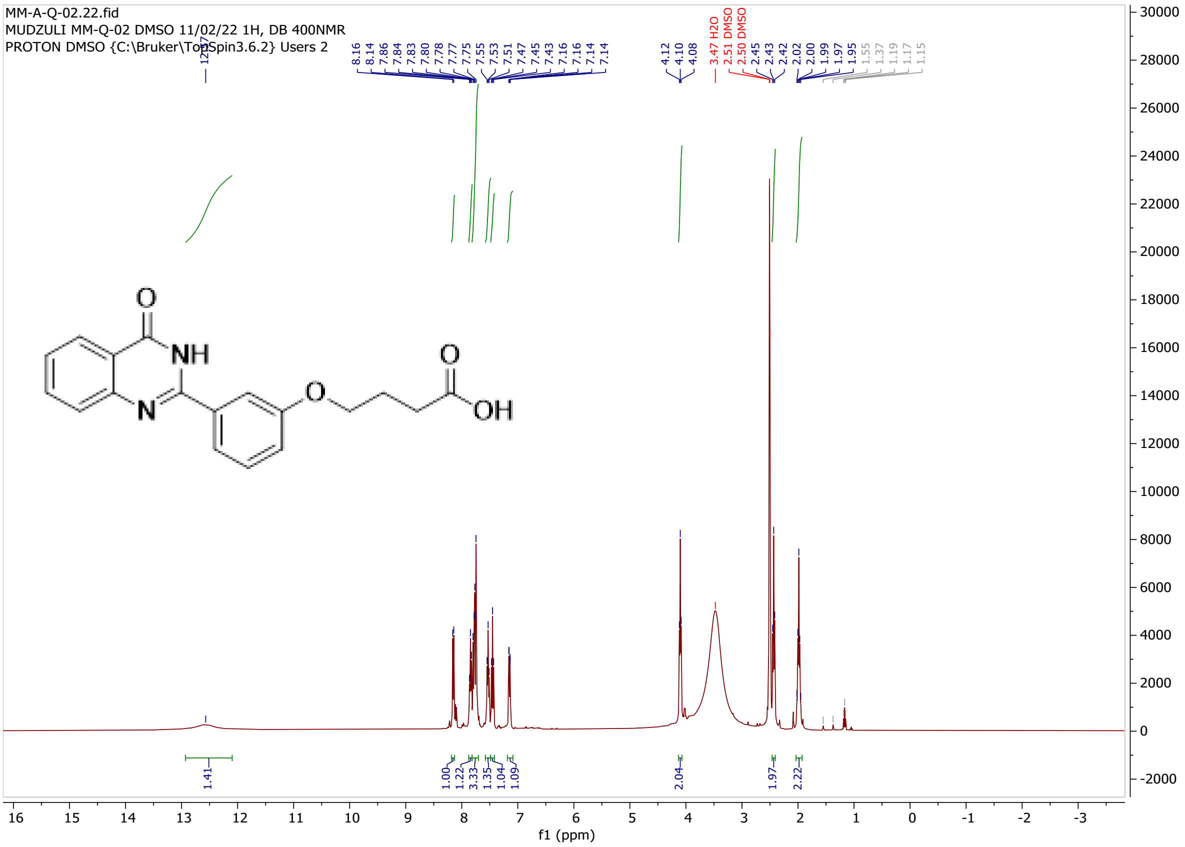

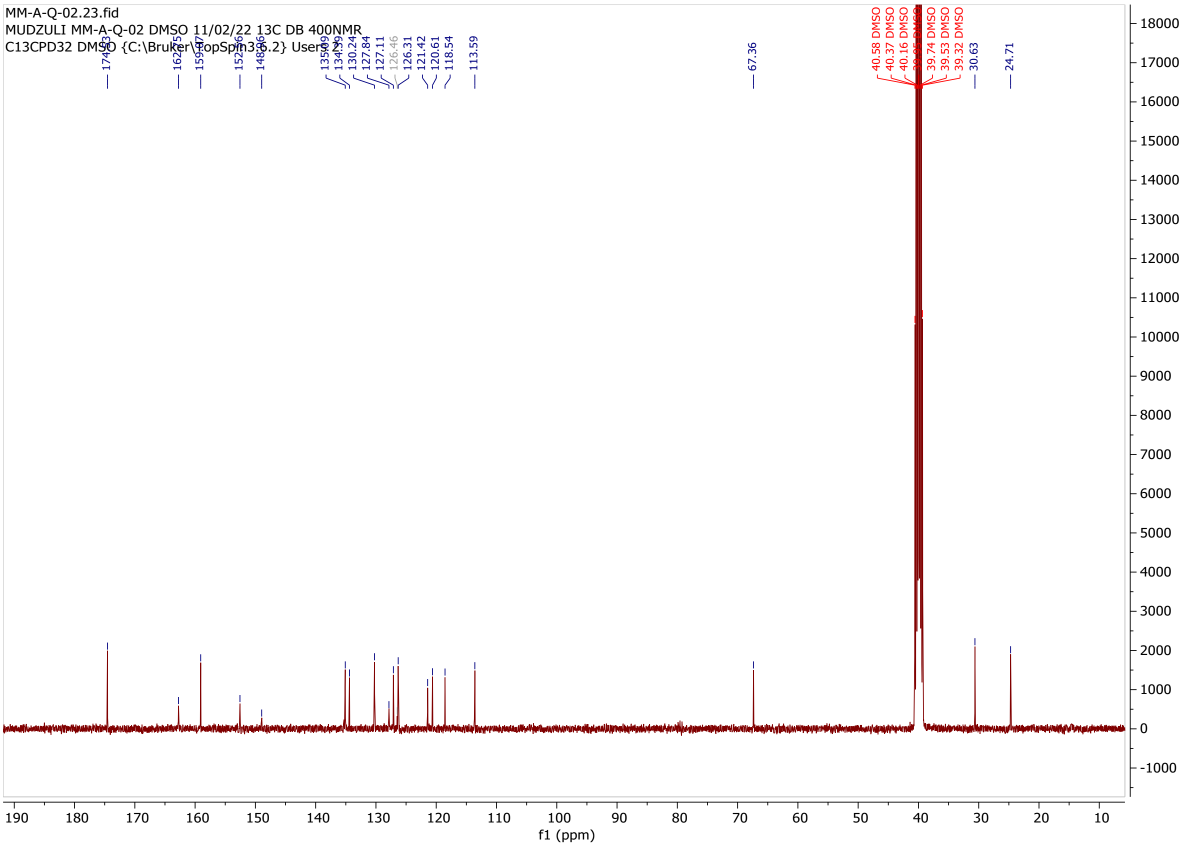

N-benzyl-4-(4-(4-oxo-3,4-dihydroquinazolin-2-yl)phenoxy)butanamide (8a)

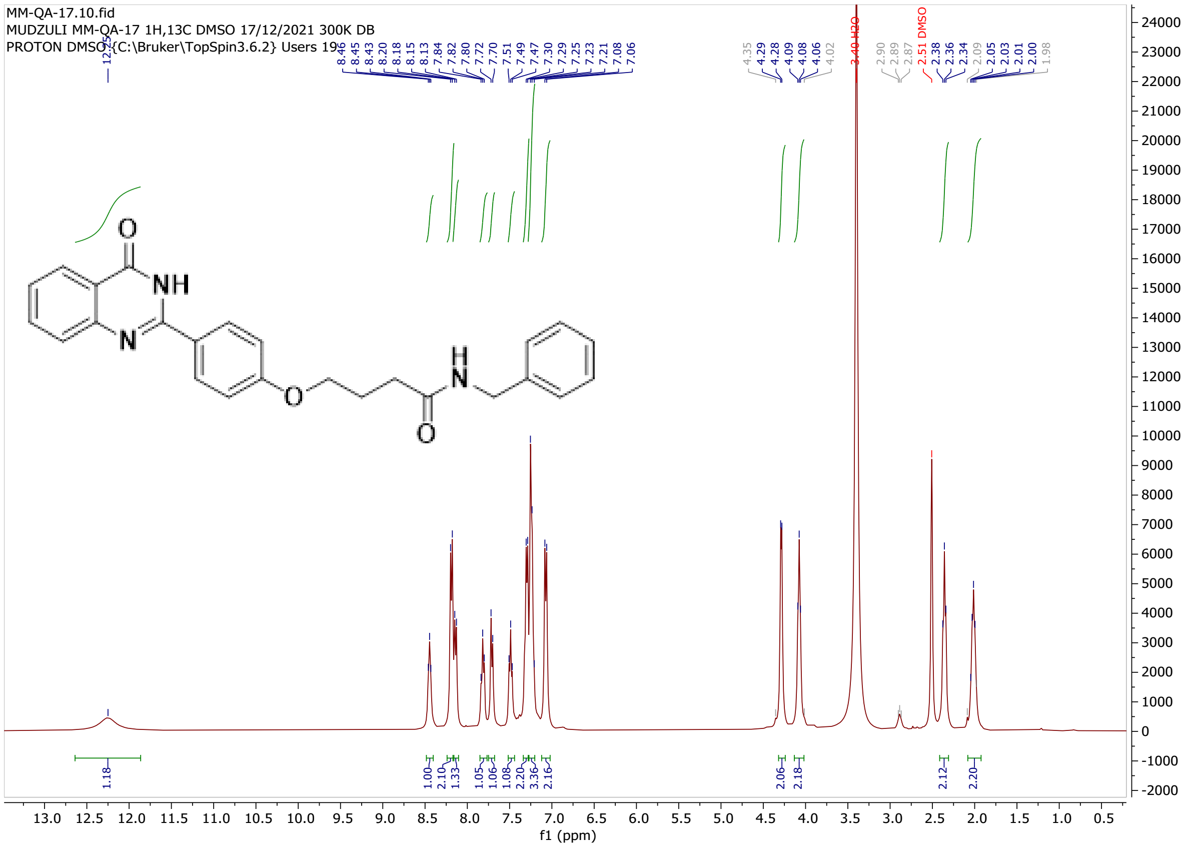
MM-QA-17.10.fid
MUDZULI MM-QA-17 1H,13C DMSO 17/12/2021 300K DB
PROTON DMSO {C:\Bruker\TopSpin3.6.2} Users 19
8.46 8.45 8.43 8.20 8.18 8.15 8.13 7.84 7.82 7.80 7.72 7.70 7.51 7.49 7.47 7.30 7.29 7.25 7.23 7.21 7.08 7.06
4.35 4.29 4.28 4.09 4.08 4.06 4.02
3.40 H2O
2.90 2.89 2.87
2.51 DMSO
2.38 2.36 2.34 2.09 2.05 2.03 2.01 2.00 1.98
1.18 1.00 2.10 1.33 1.05 1.06 1.08 2.20 3.36 2.16 2.06 2.18 2.12 2.20
NH
N
O
H
f1 (ppm)

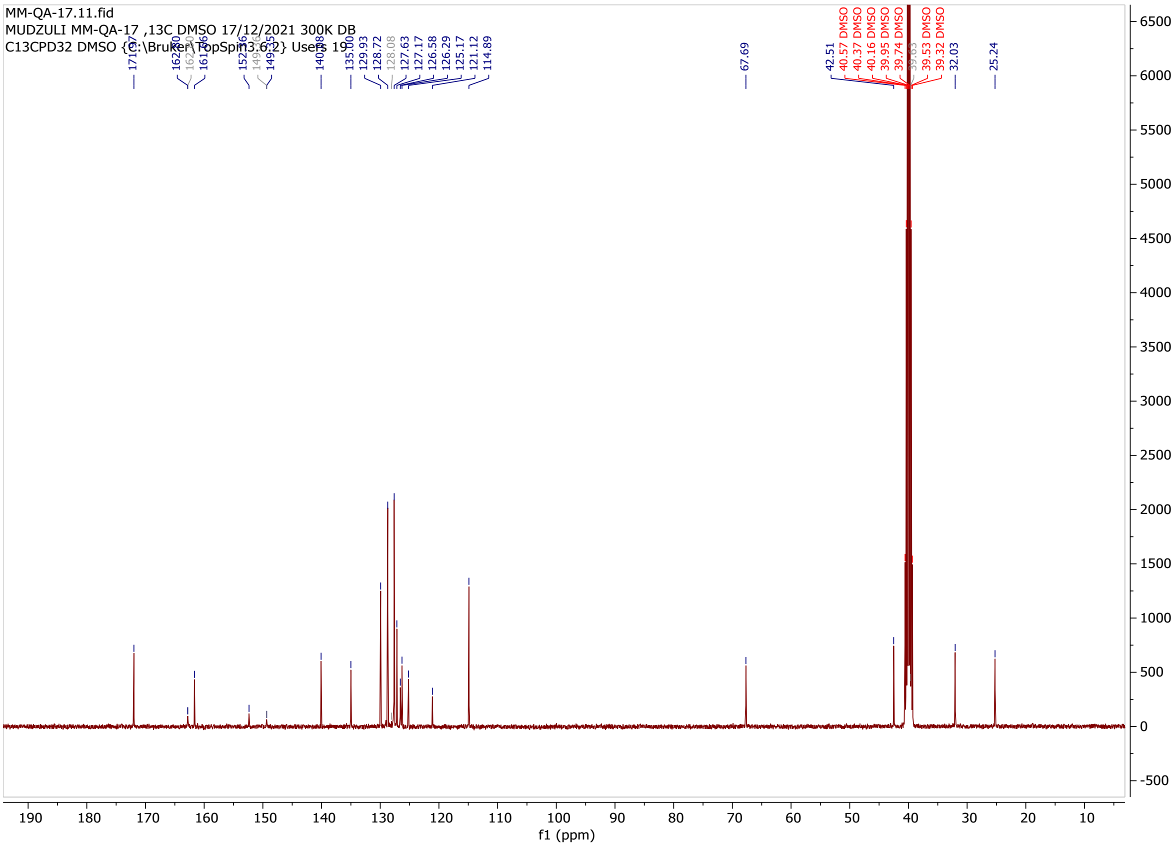
MM-QA-17.11.fid
MUDZULI MM-QA-17 ,13C DMSO 17/12/2021 300K DB
C13CPD32 DMSO {C:\Bruker\TopSpin3.6.2} Users 19
135.00 129.93 128.72 128.08 127.63 127.17 126.58 126.29 125.17 121.12 114.89
67.69
42.51 40.57 DMSO 40.37 DMSO 40.16 DMSO 39.95 DMSO 39.74 DMSO 39.53 DMSO 39.32 DMSO
32.03
25.24
f1 (ppm)

N-(4-methoxybenzyl)-4-(3-(4-oxo-3,4-dihydroquinazolin-2-yl)phenoxy) butanamide (8b)

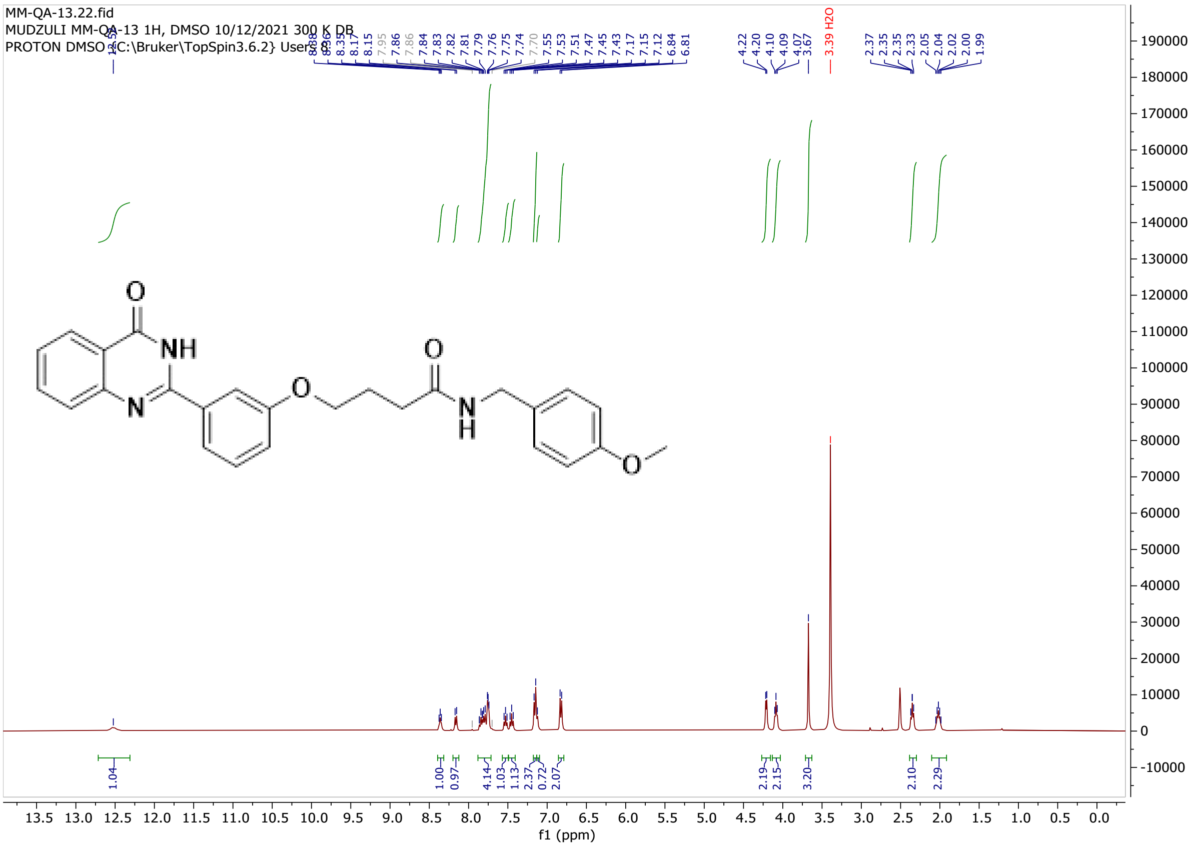

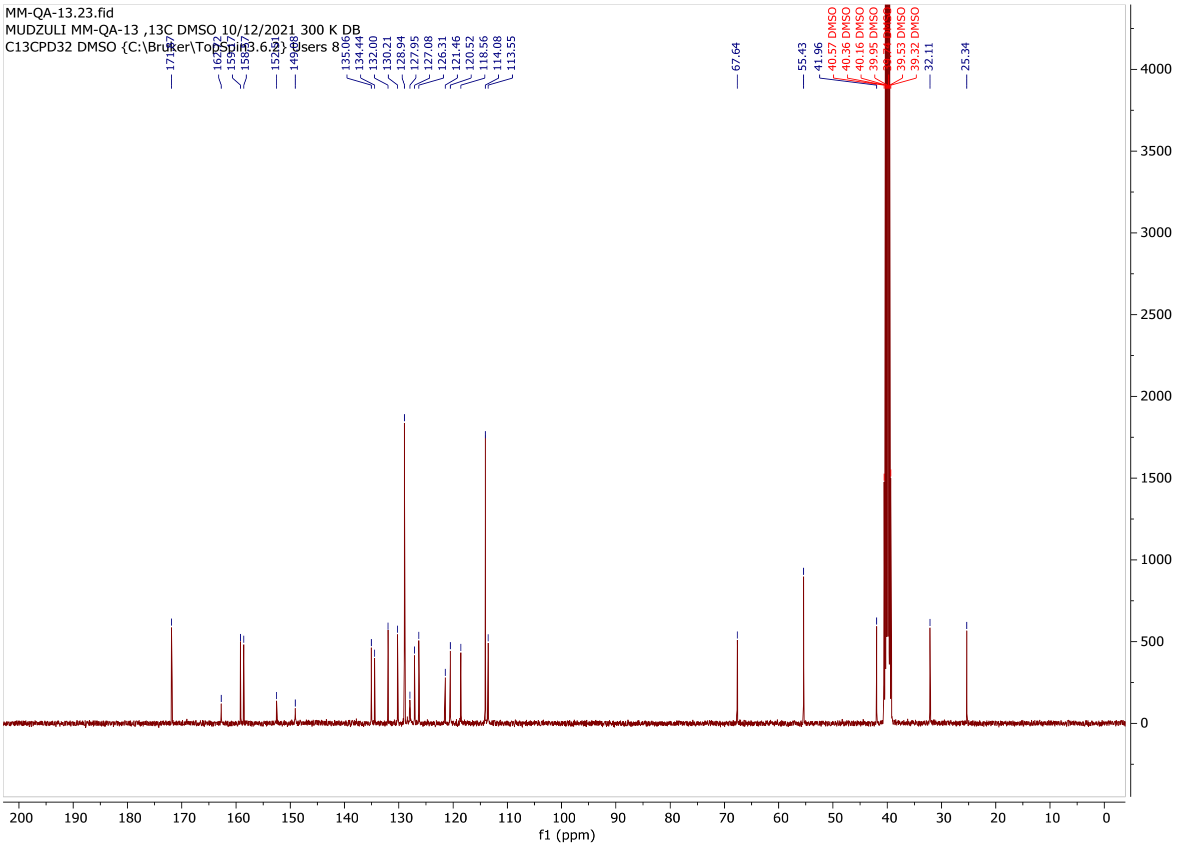

4-(2-Methoxy-4-(6-nitro-4-oxo-3,4-dihydroquinazolin-2-yl)phenoxy)-*N*-(4-methoxybenzyl)butanamide (8c)

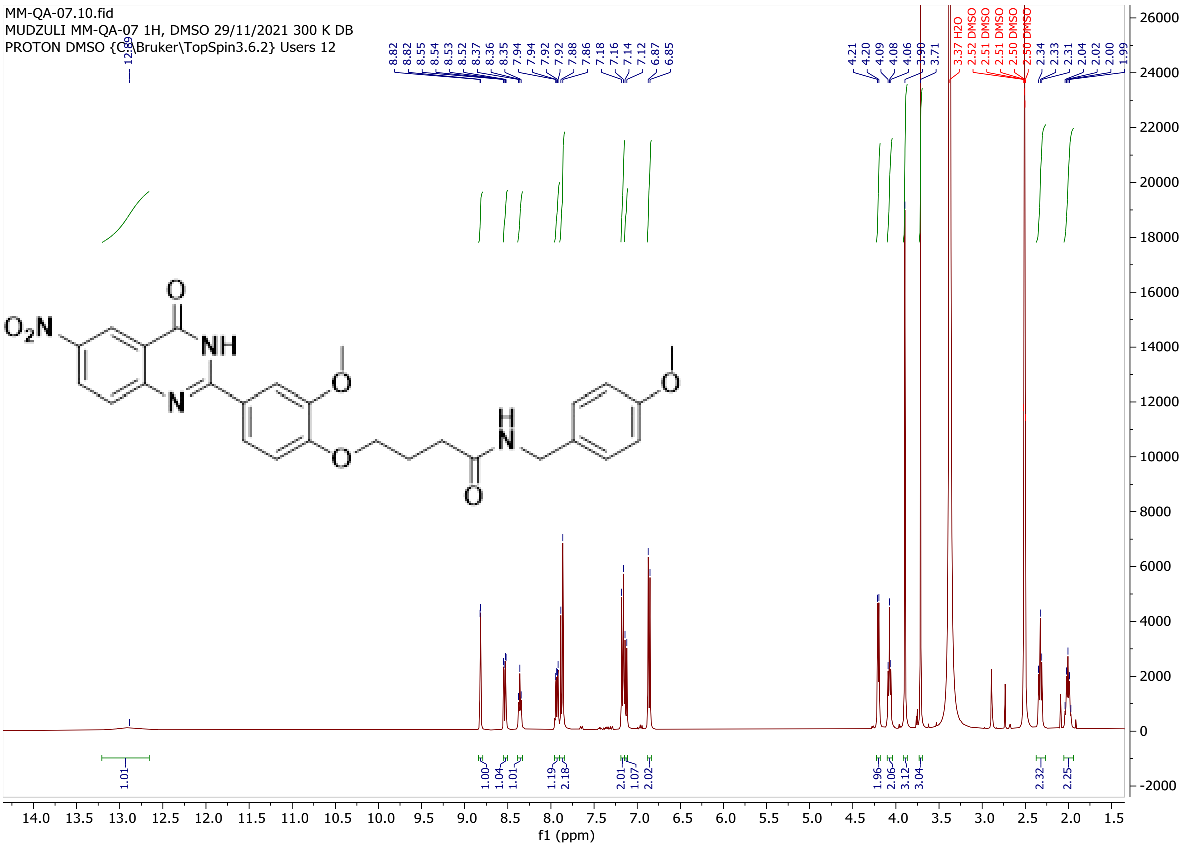

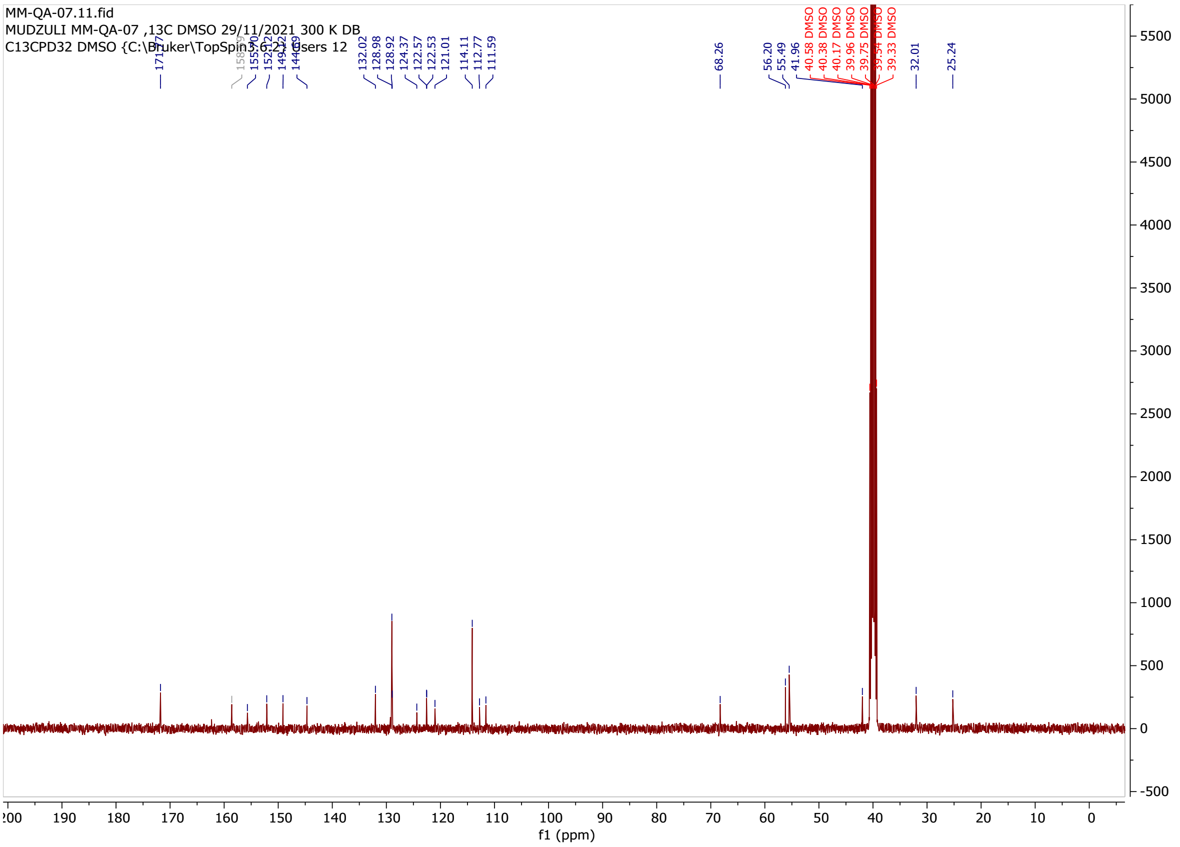

N-isopropyl-4-(4-(4-oxo-3,4-dihydroquinazolin-2-yl)phenoxy)butanamide (8f)

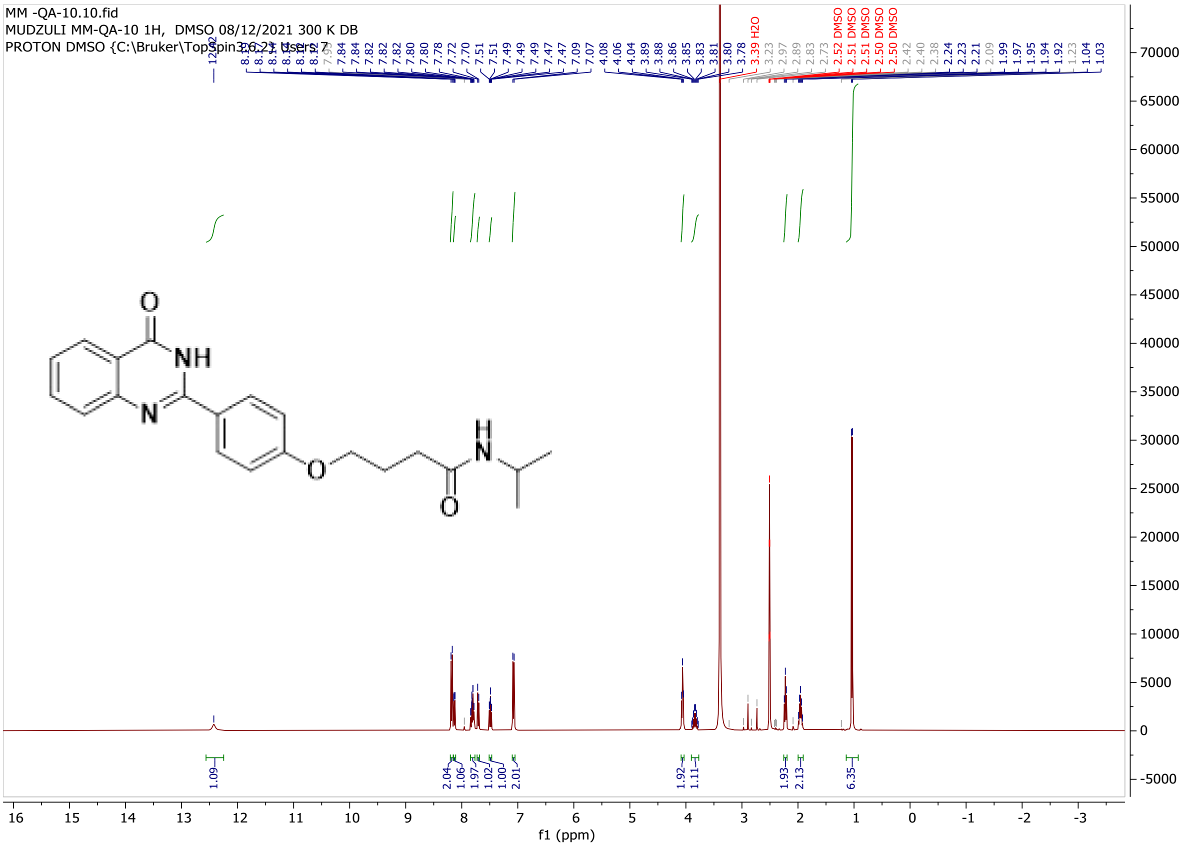

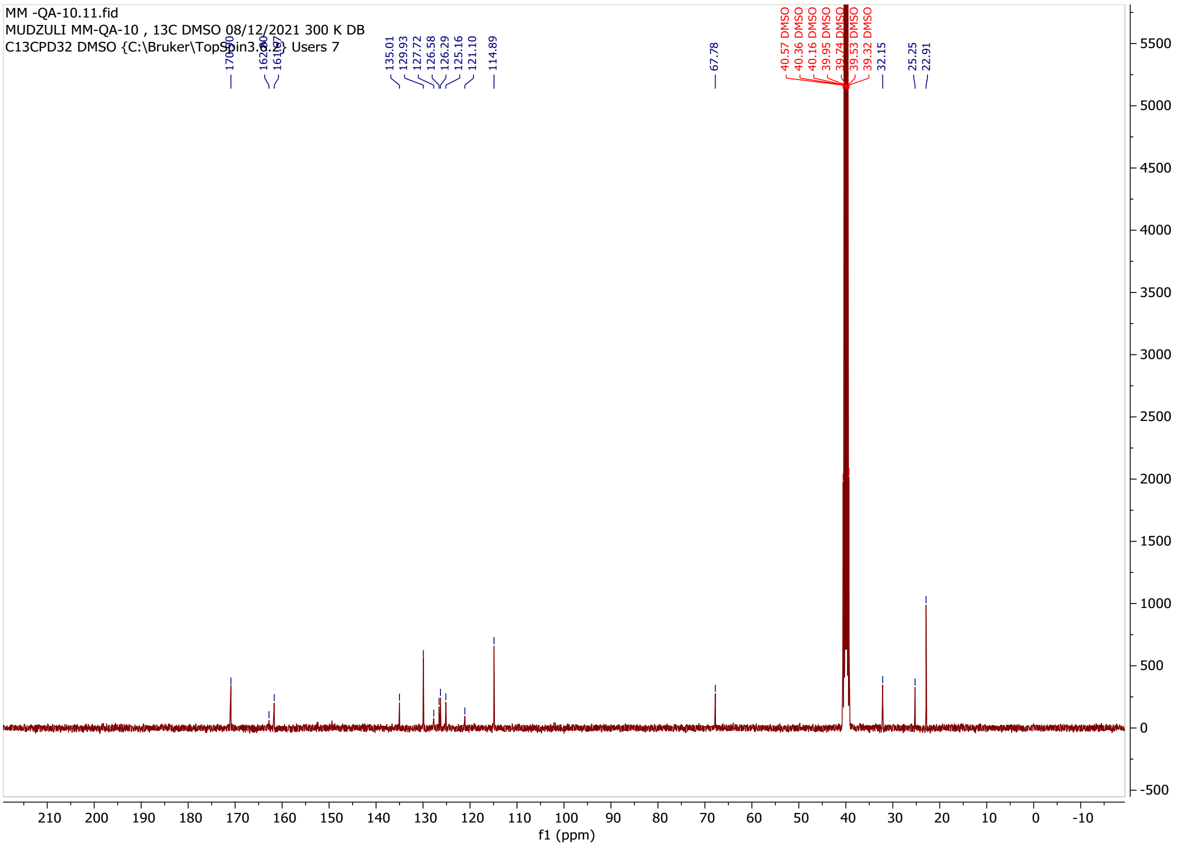

*N-i*sobutyl-4-(4-(4-oxo-3,4-dihydroquinazolin-2-yl)phenoxy)butanamide (8j)

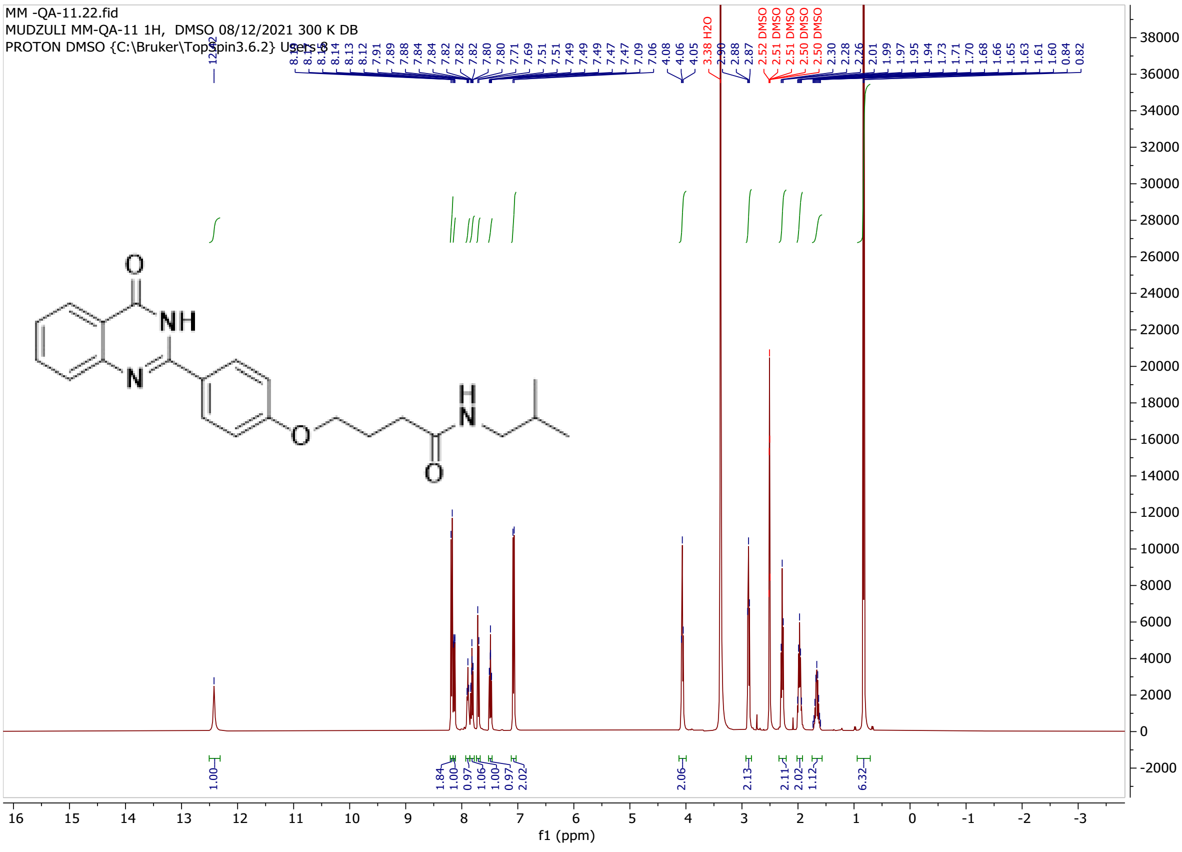

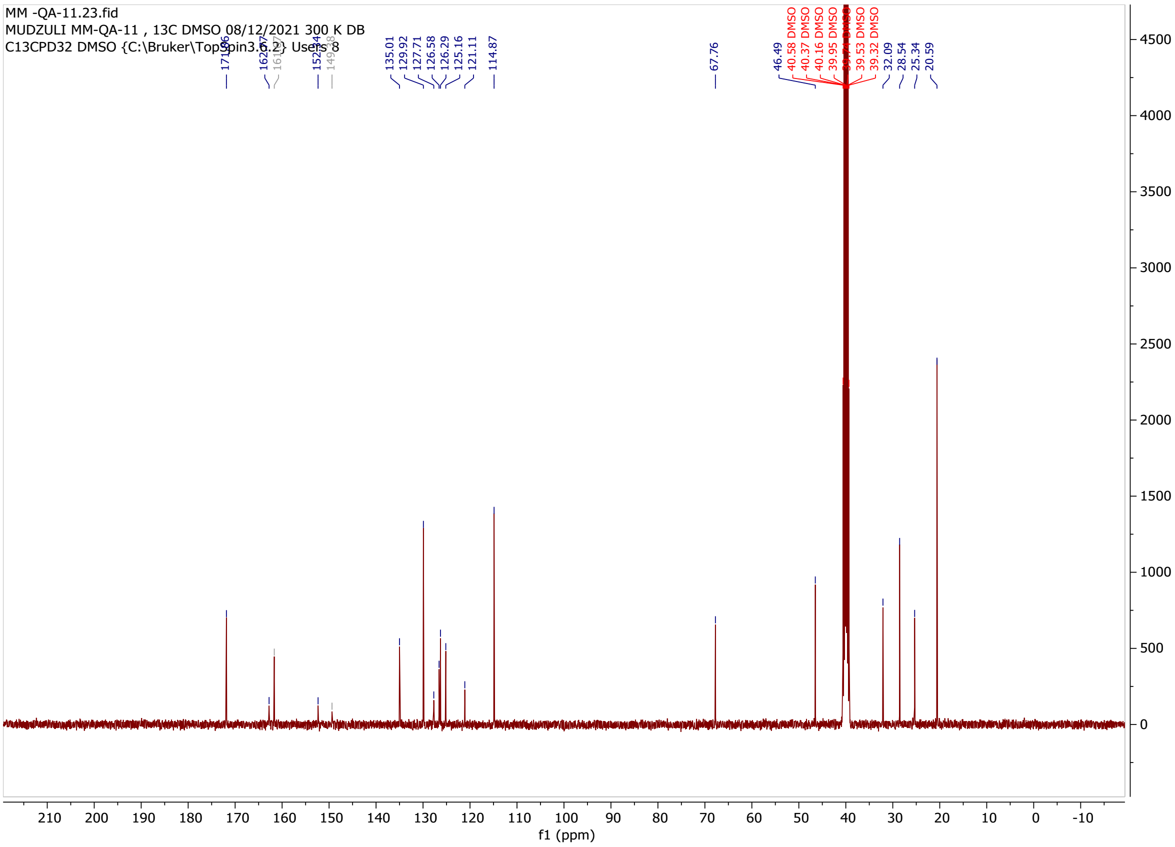

_N_-(4-methoxybenzyl)-4-(3-(4-oxo-3,4-dihydroquinazolin-2-yl)phenoxy) butanamide (8b)

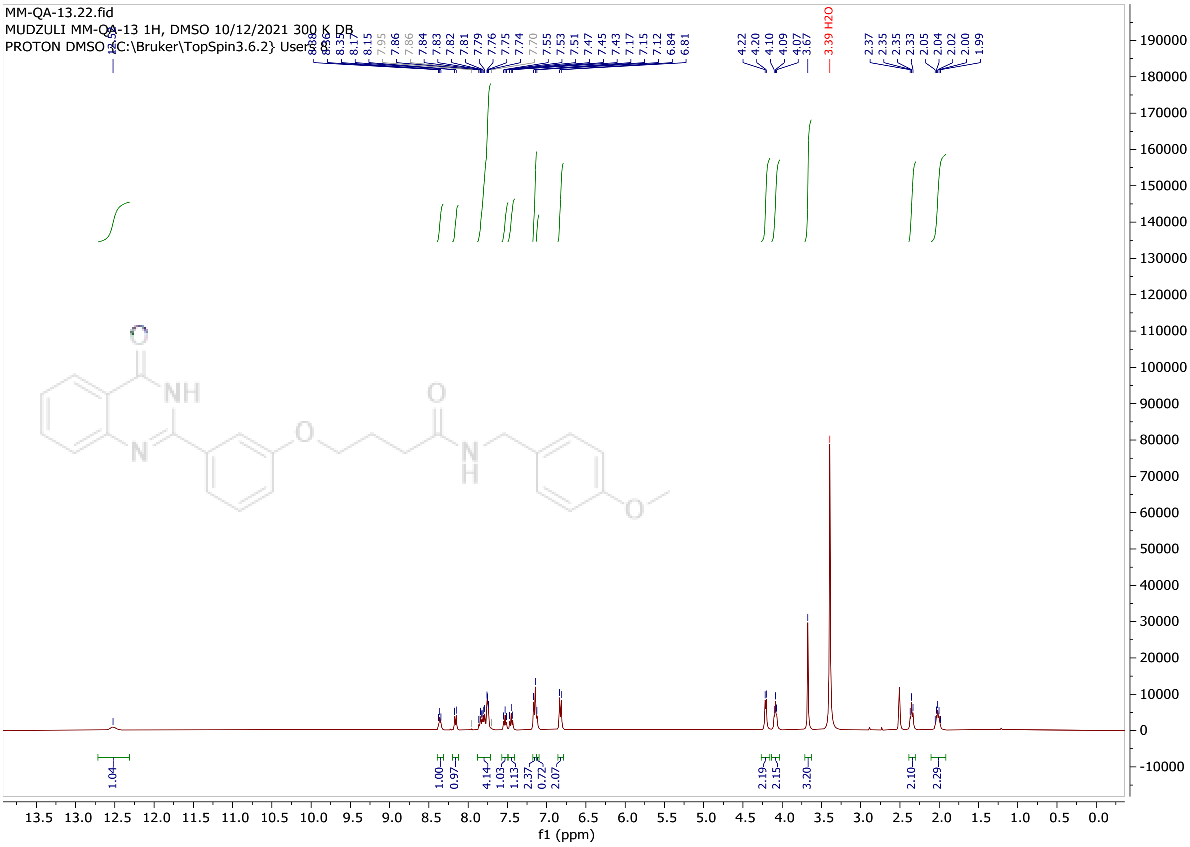
MM-QA-13.22.fid
MUDZULI MM-QA-13 1H, DMSO 10/12/2021 300 K DB
PROTON DMSO {C:\Bruker\TopSpin3.6.2} User 8
3.39 H2O
f1 (ppm)

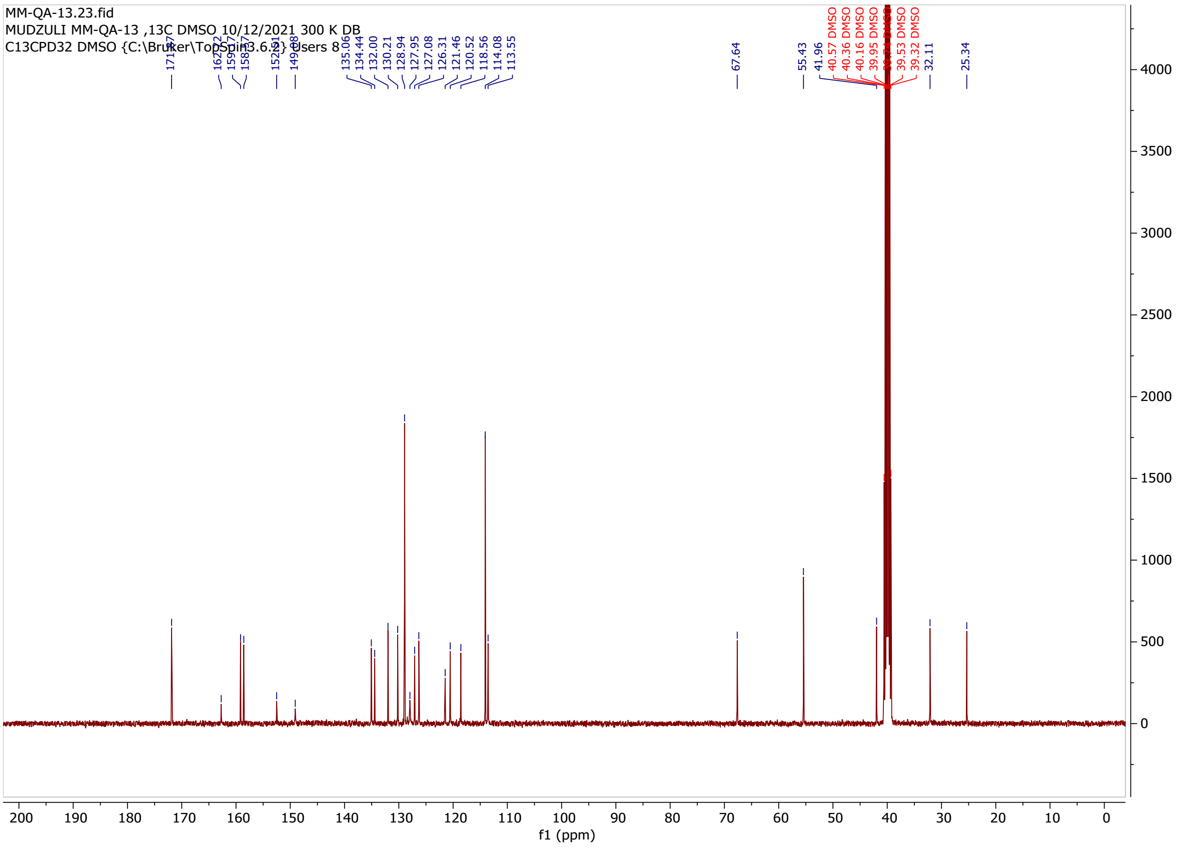
MM-QA-13.23.fid
MUDZULI MM-QA-13 ,13C DMSO 10/12/2021 300 K DB
C13CPD32 DMSO {C:\Bruker\TopSpin3.6.2} Users 8
f1 (ppm)

N-(2-ethylhexyl)-4-(4-(4-oxo-3,4-dihydroquinazolin-2-yl)phenoxy) butanamide (8p)

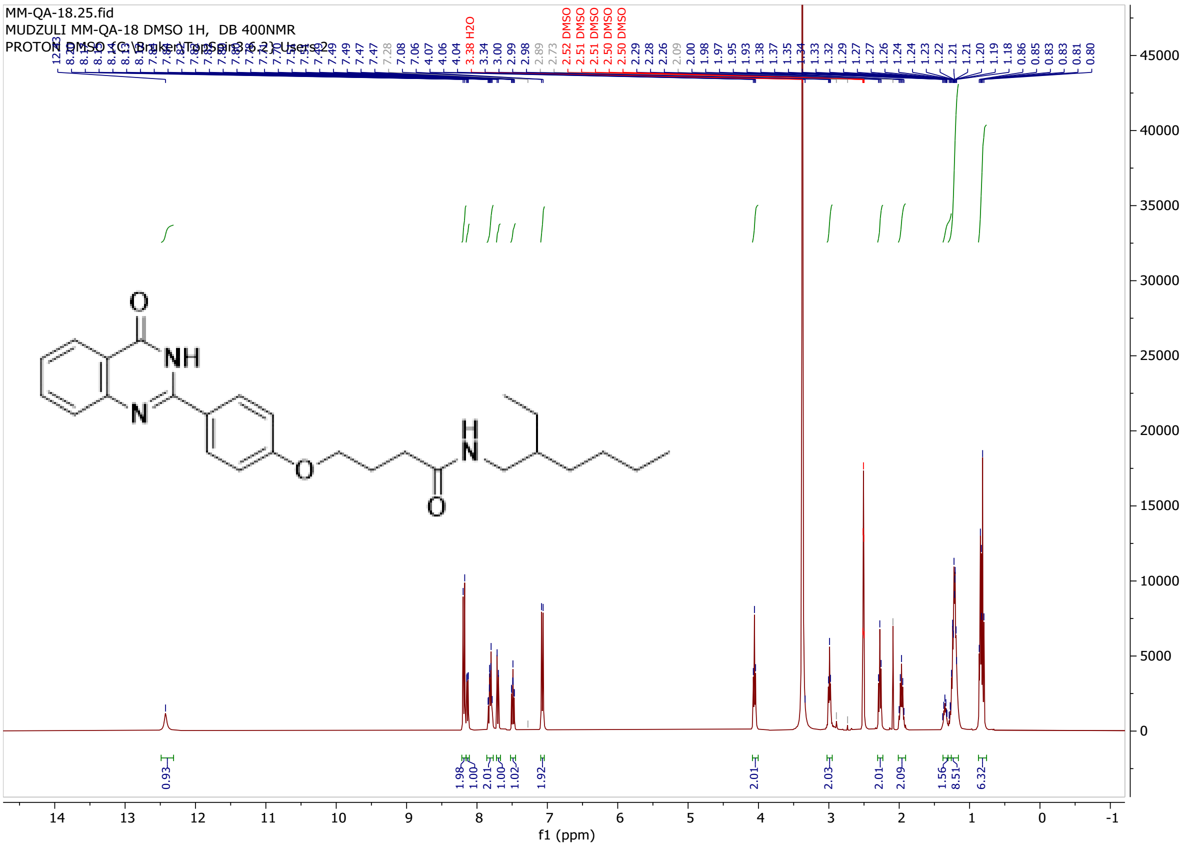

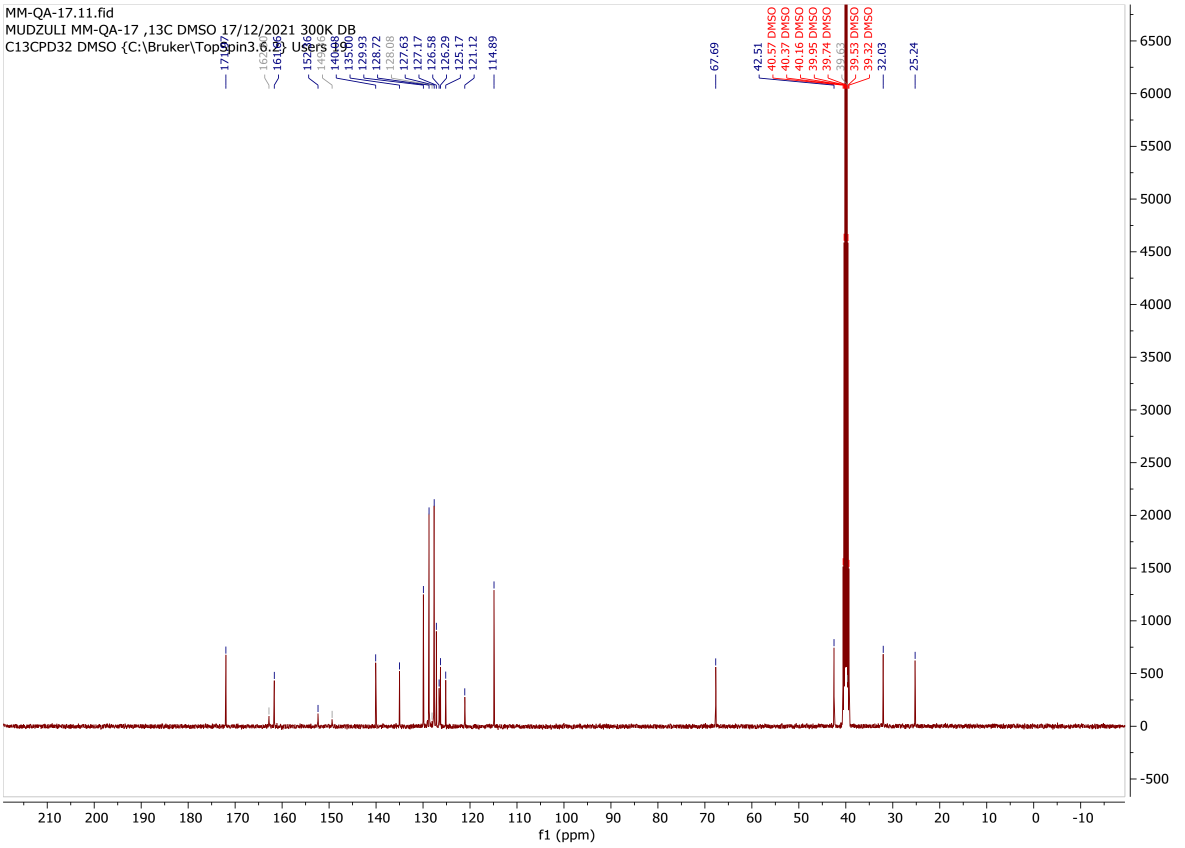

N-(3,4-dimethoxyphenethyl)-4-(3-(4-oxo-3,4-dihydroquinazolin-2-yl)phenoxy) butanamide (8q)

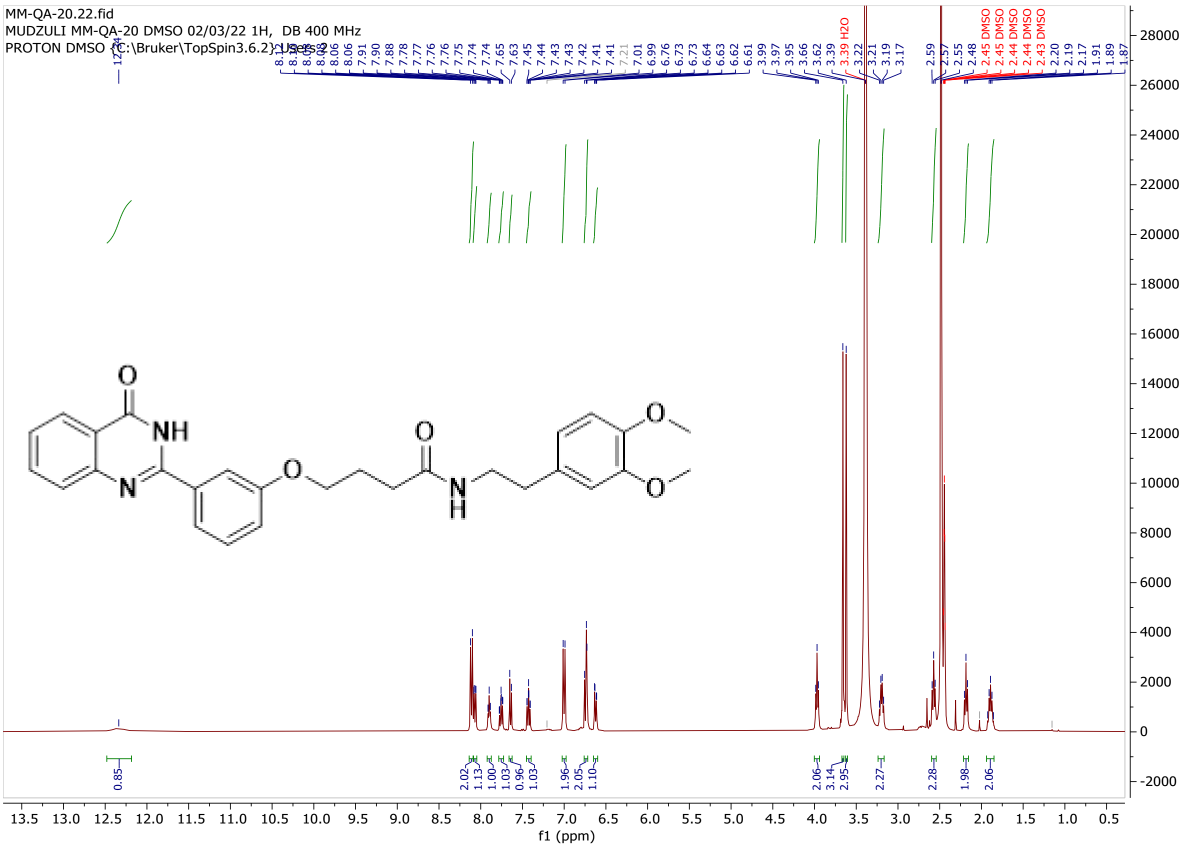

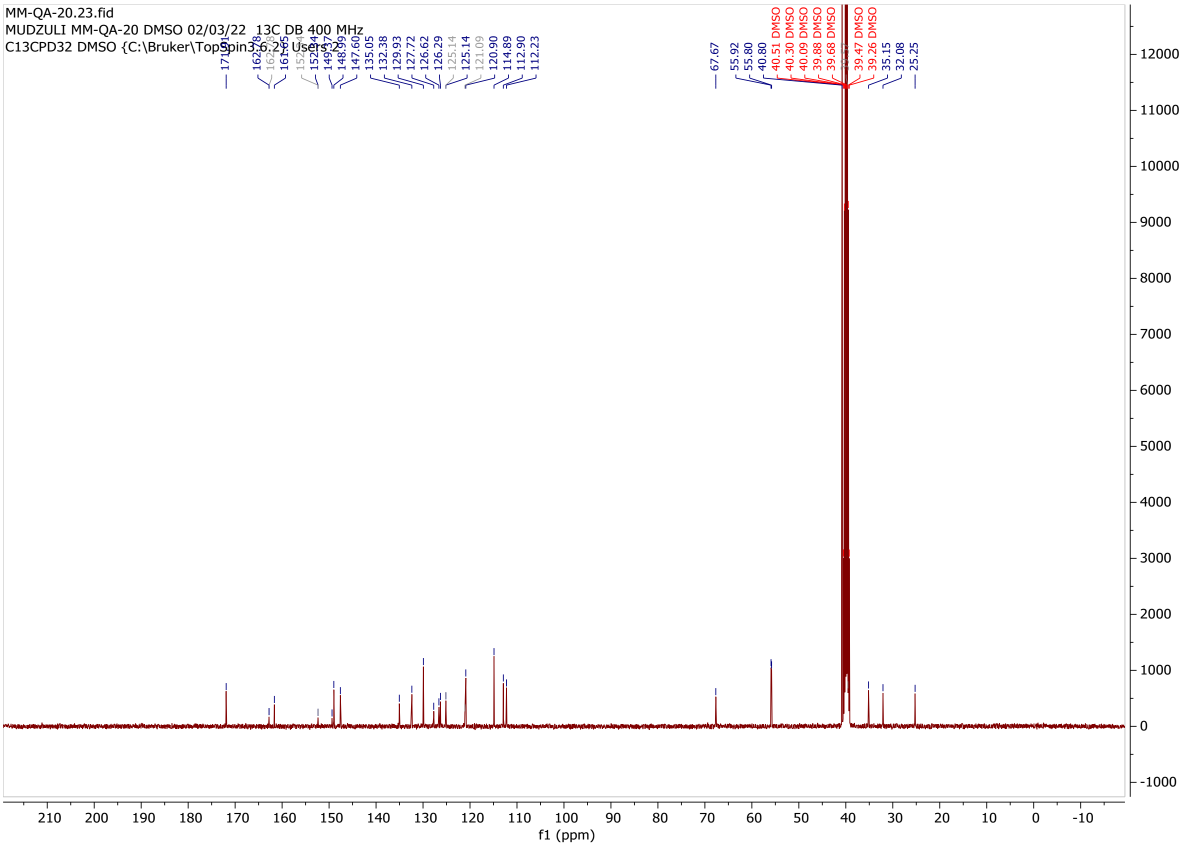

N-(4-chlorophenethyl)-4-(4-(4-oxo-3,4-dihydroquinazolin-2-yl)phenoxy) butanamide (8s)

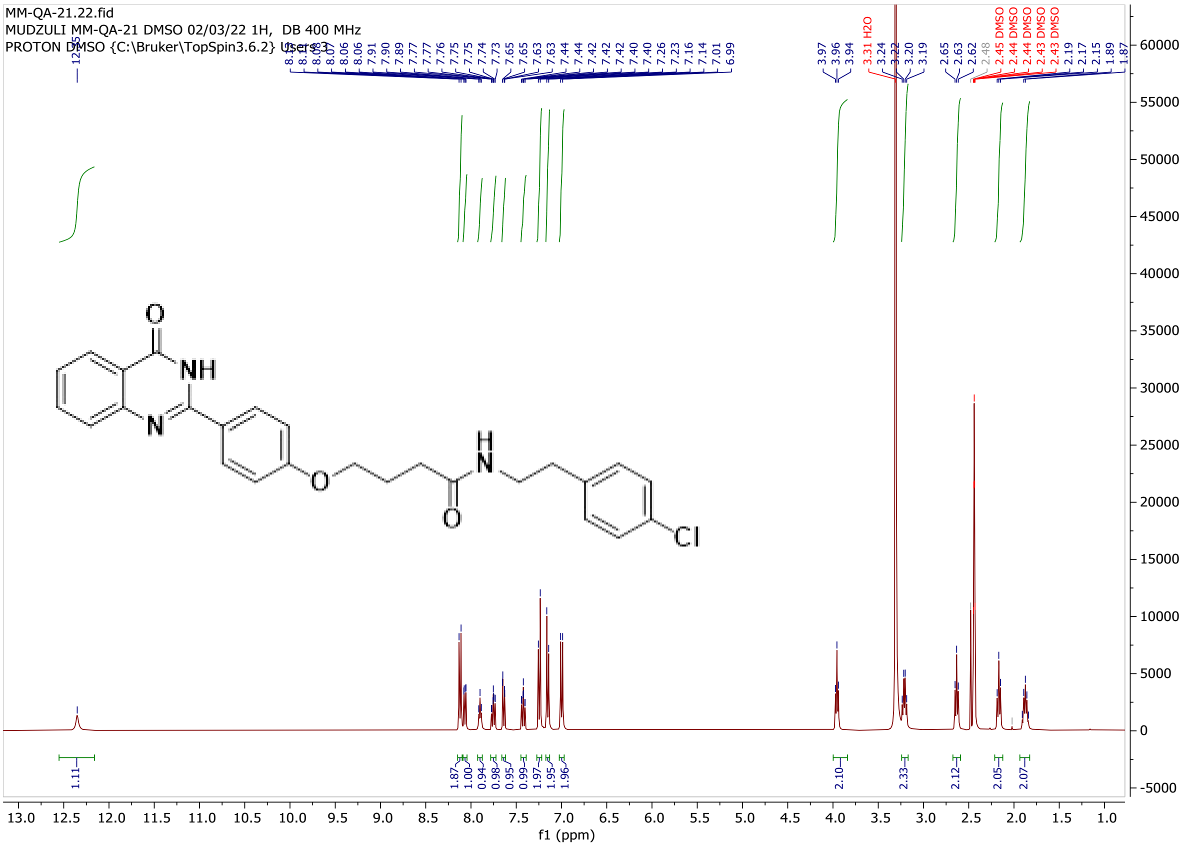

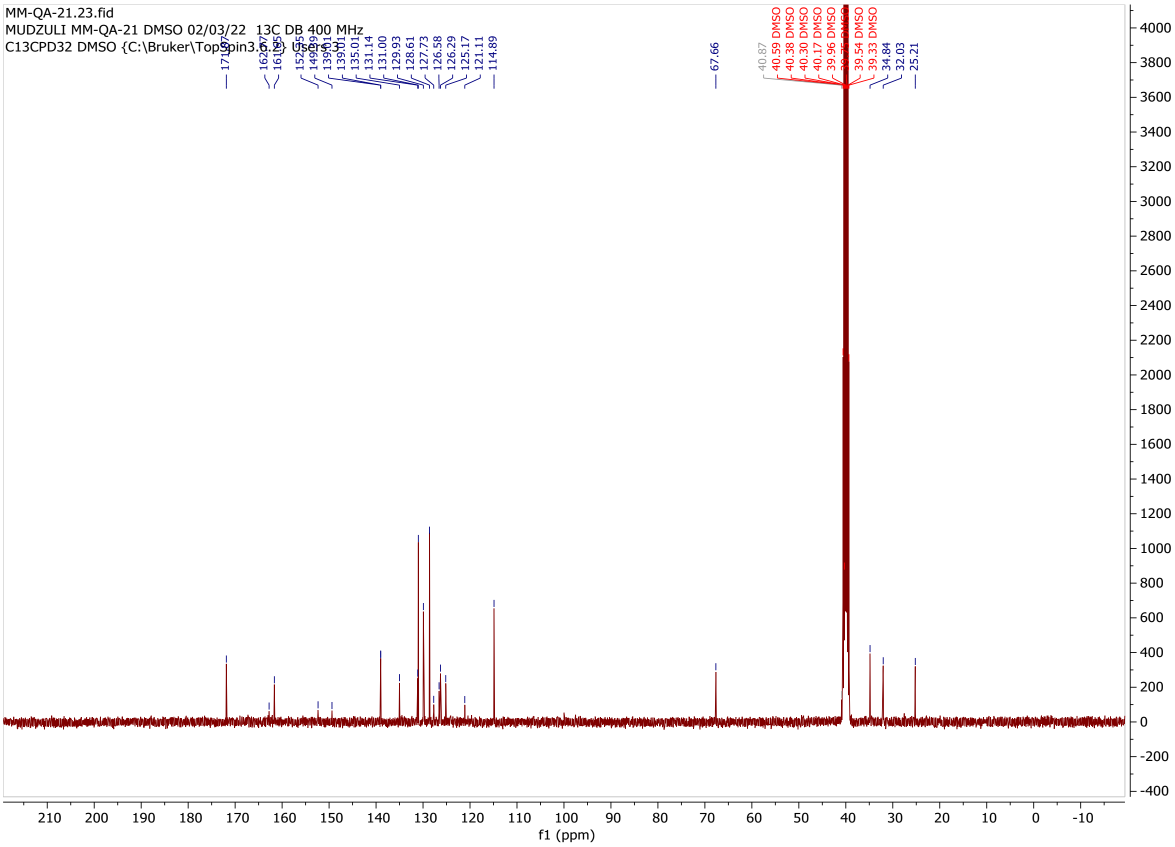
MM-QA-21.23.fid
MUDZULI MM-QA-21 DMSO 02/03/22 13C DB 400 MHz
C13CPD32 DMSO {C:\Bruker\TopSpin3.6.1} User 3
171.97
162.97
161.55
152.55
149.29
139.01
135.01
131.14
131.00
129.93
128.61
127.73
126.58
126.29
125.17
121.11
114.89
67.66
40.87
40.59 DMSO
40.38 DMSO
40.30 DMSO
40.17 DMSO
39.96 DMSO
39.54 DMSO
39.33 DMSO
34.84
32.03
25.21
f1 (ppm)
